# *Vibrio proteolyticus* DCF12.2 postbiotic modulates intestinal metabolic and immune pathways in zebrafish

**DOI:** 10.1007/s00253-026-13825-x

**Published:** 2026-04-22

**Authors:** Jorge García-Márquez, Isabel Cerezo, Rafaela Santos, Rui Magalhães, António Paulo Carvalho, Salvador Arijo, Aires Oliva-Teles, Cláudia Serra, Miguel Ángel Moriñigo

**Affiliations:** 1 Centro Interdisciplinar de Investigação Marinha e Ambiental (CIIMAR), Universidade do Porto, Terminal de Cruzeiros do Porto de Leixões, Av, General Norton de Matos s/n, Matosinhos, 4450-208 Portugal; 2 Departamento de Biologia, Faculdade de Ciências, University of Porto, Rua do Campo Alegre s/n, Ed. FC4, Porto, 4169-007 Portugal; 3 Departamento de Microbiología, Facultad de Ciencias, Universidad de Málaga, Instituto Andaluz de Biotecnología y Desarrollo Azul (IBYDA), Campus Universitario de Teatinos s/n, Málaga, 29071 Spain; 4 Centro Oceanográfico de Málaga, Instituto Español de Oceanografía (IEO), Consejo Superior de Investigaciones Científicas (CSIC), Puerto de Málaga, Málaga, 29002 Spain

**Keywords:** *Danio rerio*, Gene Set Enrichment Analysis (GSEA), Host-microbe interactions, Metabolic regulation, Postbiotics

## Abstract

**Abstract:**

Postbiotics have recently emerged as a promising alternative to probiotics in aquaculture due to their improved safety, stability, and ease of application; however, the biological effects of postbiotics derived from *Vibrio proteolyticus* remain largely unexplored. In this study, we evaluated the impact of dietary supplementation with ethanol-inactivated *V. proteolyticus* DCF12.2 on the intestinal microbiota composition and transcriptomic profile of zebrafish (*Danio rerio*). Fish were fed a control diet (CTRL group) or a diet supplemented with 1% lyophilized ethanol-inactivated *V*. *proteolyticus* DCF12.2 (VP group) for 21 days. Alpha and beta diversity analyses exhibited no significant differences in overall gut microbial composition between dietary groups, nor at the phylum or genus level. However, differential abundance analysis at the amplicon sequence variant (ASV) level identified significant differences in ASVs affiliated with the genera *Aeromonas*, *Delftia*, *Ralstonia*, *Shewanella*, and *Stenotrophomonas*, which were enriched in fish receiving the VP diet (*p* < 0.0001). Despite these ASV-level shifts, predicted microbial metabolic functions did not differ significantly between treatments. In contrast, transcriptomic analysis via Gene Set Enrichment Analysis (GSEA) detected a clear host response: a significant downregulation of the cytokine–cytokine receptor interaction pathway and the upregulation of pathways related to nutrient metabolism, oxidative phosphorylation, and cellular biosynthesis in fish fed the VP diet (adjusted *p* < 0.05). These results suggest that dietary supplementation with postbiotics from *V. proteolyticus* DCF12.2 modulates intestinal metabolic and immune pathways without inducing major restructuring of the gut microbiota composition. Collectively, these findings provide new insights into host–postbiotic interactions and support the potential application of postbiotics from *V. proteolyticus* DCF12.2 as functional feed additives for sustainable aquaculture.

**Key points:**

• *Postbiotic diet did not alter gut microbial diversity but induced ASV-level differences*

• *Postbiotic diet upregulated nutrient metabolism, oxidative phosphorylation, and cellular biosynthesis*

• *Cytokine–cytokine* *receptor signaling was downregulated by the postbiotic diet*

**Supplementary Information:**

The online version contains supplementary material available at 10.1007/s00253-026-13825-x.

## Introduction

Advancements in functional feeds have promoted the development of health-promoting dietary strategies in aquaculture, among which probiotics have received considerable attention. Probiotics are defined as “live microorganisms that, when administered in adequate amounts, can confer a health benefit on the host” (Hill et al. 2014), and numerous studies have demonstrated their potential to enhance the health of both humans and farm animals (Kerry et al. 2018; Abd El-Hack et al. 2020; Ringø et al. 2020). Despite these benefits, several limitations restrict their practical application in aquafeeds, including reduced viability during feed processing and storage, inconsistent colonization and persistence in the gut, and potential biosafety concerns such as the horizontal transfer of virulence or antibiotic resistance genes (Choudhury and Kamilya 2019). To overcome these constraints, postbiotics have emerged as an effective alternative. Postbiotics are defined as preparations of inanimate microorganisms and/or their components that confer health benefits on the host (Salminen et al. 2021). Compared to probiotics, postbiotics offer several practical advantages, including improved handling, storage stability, safety, and minimal interactions with feed ingredients (de Almada et al. 2016; Vinderola et al. 2023). Increasing evidence indicates that postbiotics can modulate immune responses, intestinal microbiota, and host physiology in aquatic organisms (Yang et al. 2014; Giri et al. 2015, 2020; Van Nguyen et al. 2019). However, despite growing interest in postbiotics, their mechanisms of action in fish, particularly regarding microbiota–host interactions and host metabolic responses, remain poorly understood.

Building on this concept, microorganisms previously identified as probiotics may also serve as valuable sources of postbiotic preparations, as their inactivated cells and cellular components can retain bioactive properties that modulate host physiology. In this context, a *Vibrio proteolyticus* DCF12.2 strain was previously isolated from the intestine of healthy wedge sole (*Dicologlossa cuneata*), and characterized for its promising probiotic attributes (Medina et al. 2020). The strain exhibited antagonistic activity against fish pathogens, absence of virulence towards the host, and stability during storage. Moreover, *V. proteolyticus* DCF12.2 displayed a broad enzymatic profile, including lecithinase, gelatinase, caseinase, amylase, and lipase activities, suggesting a potential contribution to digestive processes and nutrient utilization in fish (Medina et al. 2020). Importantly, passage through the gastrointestinal tract did not compromise the viability of the strain (Medina et al. 2023). Furthermore, dietary administration of this bacterium in *Solea senegalensis* diets enhanced immune-related gene expression, increased the production of specific antibodies with cross-reactivity against *Photobacterium damselae* subsp. *piscicida* and *Vibrio harveyi*, and improved resistance against these pathogens, highlighting its potential as a functional microbial candidate for aquaculture applications (Medina et al. 2023).

Despite these well-established probiotic properties, the potential of *V. proteolyticus* DCF12.2 as a postbiotic remains insufficiently characterized. Recent studies using lyophilized ethanol-inactivated *V. proteolyticus* DCF12.2 cells modulated nutrient utilization, muscle fatty acid profile, metabolic responses, digestive physiology, intestinal microbiota, and gene expression when included in an experimental diet for the thicklip grey mullet, *Chelon labrosus* (García-Márquez et al. 2023a, b). The use of ethanol-inactivated bacterial cells allows the evaluation of postbiotic effects independently of bacterial colonization or proliferation, thereby enabling the identification of host responses triggered by structural cell components or residual bioactive metabolites. However, the molecular mechanisms through which inactivated *V. proteolyticus* cells interact with the host, particularly regarding the coordination between gut microbial communities and host transcriptional responses, remain largely unknown. Advanced molecular approaches, such as transcriptomics and high-resolution microbiota profiling, are required to clarify how postbiotic preparations influence host metabolic and immune pathways.

To further elucidate the biological effects of postbiotics derived from this strain, it is necessary to investigate the interaction between inactivated bacterial preparations, host transcriptional responses, and intestinal microbial communities. In this context, the zebrafish (*Danio rerio*) represents a well-established vertebrate model for studying host–microbiota interactions, intestinal physiology, and immune responses due to its genetic tractability, well-characterized microbiome, and extensive availability of molecular tools (Roeselers et al. 2011; Zhong et al. 2022; Thormar et al. 2024). Moreover, zebrafish have been widely used in nutritional and functional feed studies to explore the mechanistic effects of dietary interventions before their validation in aquaculture species (Ulloa et al. 2011, 2014). Therefore, the present study aimed to evaluate whether a short-term dietary supplementation with ethanol-inactivated *V. proteolyticus* DCF12.2 modulates intestinal microbiota composition and host transcriptional pathways in zebrafish. We hypothesized that short-term feeding with postbiotics derived from this strain could induce early intestinal responses, influencing gut microbial community and host metabolic pathways, thereby providing mechanistic insights into their potential application as functional feed additives for sustainable aquaculture.

## Materials and methods

### Postbiotic production

The strain *V. proteolyticus* DCF12.2 (CECT 9300), originally obtained from the intestinal tract of healthy wedge sole (*D. cuneata*) (Medina et al. 2020), was grown on tryptic soy agar (TSA; Oxoid Ltd., Basingstoke, UK) supplemented with 1.5% NaCl. Cultures were incubated at 22 °C for 24 h.

Following incubation, bacterial biomass was collected from the agar surface, resuspended in sterile phosphate-buffered saline (PBS), and combined into a single suspension. Cells were harvested by centrifugation at 6000 × g for 15 min at 4 °C. The resulting pellet was resuspended in PBS and adjusted to a final concentration of 1 × 10^11^ CFU mL⁻^1^, corresponding to an optical density at 600 nm (OD_600_) of 1.0. Viable cell counts were confirmed by plating on TSA and performing colony enumeration.

For inactivation, the bacterial suspension was treated with ethanol, and the volume of ethanol was adjusted to achieve a final concentration of 70% (v/v) (García-Márquez et al. 2023a). Cells were maintained at room temperature for 5 min, then stored at −80 °C for 12 h, and subsequently freeze-dried for 48 h at −85 °C using a LyoQuest laboratory lyophilizer (Telstar, Tokyo, Japan). Inactivation was confirmed by plating aliquots of the treated bacterial suspension on TSA plates and incubating at 22 °C for 48 h. No colony growth was observed under these conditions. This inactivation protocol was previously validated by our research group (García-Márquez et al. 2023a), demonstrating complete loss of viability while preserving enzymatic activities (protease, gelatinase, lipase, and amylase) associated with *V. proteolyticus* DCF12.2 after ethanol treatment.

### Experimental diets

Two experimental diets were formulated: a plant feedstuff-based practical diet (control diet; CTRL) and the CTRL supplemented with 1% of lyophilized *V. proteolyticus* DCF12.2 (VP diet) (Table 1). The ingredients were well mixed before fish oil and choline chloride were added to the blend. All the ingredients were mixed for 15 min, and water was added to obtain a homogeneous dough. The mixtures were then pelleted in a laboratory pellet mill (California Pellet Mill, Crawfordsville, IN, USA) and dried in an oven at 40 °C for 48 h. The dried pellets were then crushed and sieved to obtain granules of 200–400 µm. Diets were kept in sealed plastic bags at −20 °C until use.

**Table 1:** Ingredient composition of the experimental diets (g kg^−1^ dry weight)

	CTRL	VP
Fish meal^a^	605	605
CSPS^b^	30	30
Maize starch^c^	315	305
Fish oil^d^	15	15
Mineral mix^e^	10	10
Vitamin mix^f^	10	10
Choline chloride (50%)^g^	5	5
Binder^h^	10	10
*V. proteolyticus* biomass	0	10

^a^Pesquera Centinela, Steam Dried LT, Chile (CP: 70.3%; CL: 12%). Sorgal, S.A. Ovar, Portugal

^b^Sopropèche, Wimille, France (CP: 78%; CL: 8%)

^c^C-Gel Instant – 12016, Cerestar, Mechelen, Belgium

^d^Cod liver oil

^e^Vitamin premix (mg/kg diet): retinol, 18,000 (IU/kg diet); calciferol, 2000 (IU/kg diet); alpha-tocopherol, 35; menadione sodium bis., 10; thiamin, 15; riboflavin, 25; Ca pantothenate, 50; nicotinic acid, 200; pyridoxine, 5; folic acid, 10; cyanocobalamin, 0.02; biotin, 1.5; ascorbyl monophosphate, 50; inositol, 400

^f^Minerals premix (mg/kg diet): cobalt sulfate, 1.91; copper sulfate, 19.6; iron sulfate, 200; sodium fluoride, 2.21; potassium iodide, 0.78; magnesium oxide, 830; manganese oxide, 26; sodium selenite, 0.66; zinc oxide, 37.5; dicalcium phosphate, 8.02 (g/kg diet); potassium chloride, 1.15 (g/kg diet); sodium chloride, 0.4 (g/kg diet)

^g^Sorgal, S.A. Ovar, Portugal

^h^Aquacube, Agil, UK

### Ethics statement

The experiment was conducted under the direction of accredited scientists, following the recommendations of the Federation of European Laboratory Animal Science Associations (FELASA) for category C, and in accordance with the European Union Directive 2010/63/EU on the protection of animals used for scientific purposes. The experimental protocol was approved by the Animal Welfare and Ethics Body of the Interdisciplinary Centre of Marine and Environmental Research (ORBEA-CIIMAR) under reference ORBEA_CIIMAR_27_2019.

### Zebrafish larvae general care

Zebrafish embryos were obtained through mass spawning from a wild-type broodstock (12–18 months) maintained in a glass tank at the CIIMAR facilities. Embryos (until hatching) and newly-hatched larvae (until yolk sac depletion) were incubated in glass vessels with dechlorinated water. Following yolk sac depletion, larvae were transferred to a recirculating water system similar to that described by Charlon and Bergot (1984) and fed twice a day with ZebraFeed (Sparos, Olhão, Portugal; 60% crude protein, 12% lipids) until reaching the desired size for the experimental trial. Throughout all stages, key environmental parameters remained at the values or within the reference ranges recommended for the species (Aleström et al. 2020; Hammer 2020): temperature 26–29 °C, photoperiod of 14 h light/10 h dark, dissolved oxygen 6–8 mg L⁻^1^, pH 7–8, ammonia < 0.1 mg L⁻^1^, nitrite < 0.3 mg L⁻^1^, and nitrate < 0.25 mg L⁻^1^.

### Feeding trial and sampling

The feeding trial was conducted in a recirculating water system equipped with 10-L plastic tanks, maintained at a temperature of 28 ± 1 °C, and with a photoperiod of 14 h light/10 h dark. A group of 90 zebrafish juveniles (initial body weight (IBW): 0.05 ± 0.01 g) was transferred to 6 tanks (*n* = 15 per tank). The feeding trial followed a randomized complete block design, with the position within the experimental system (the two ends and the central area) serving as the blocking factor to minimize potential bias from spatial gradients. Each experimental diet (CTRL and VP) was allocated to three tanks (*n* = 3), with one replicate per diet randomly assigned to each block. Fish were fed the experimental diets by hand twice daily until apparent satiation, 6 days a week, for 21 days. During the trial, nitrogenous compounds remained at concentrations below the recommended safety levels mentioned above, and pH remained at 7.2. At the end of the trial, 7 fish per tank (21 per treatment) were individually weighed and euthanized with an overdose of tricaine methanesulphonate (MS-222, 300 mg L^−1^). Intestines were collected under aseptic conditions and stored in RNA later at −80 °C until evaluation of microbiota (3 fish per tank, 9 per condition) and RNA-seq (4 fish per tank, 12 per condition) analyses.

### Characterization of the intestinal microbiota

DNA from gut samples was extracted following a protocol based on saline precipitation (Martínez et al. 1998) with minor modifications (Tapia-Paniagua et al. 2010). Briefly, the samples were mixed with 300 μL of resuspension buffer (0.1 M Tris–HCl, 0.01 M NaCl, 0.1 M EDTA, pH 8) and 300 μL of lysis buffer (0.1 M Tris–HCl, 0.1 M EDTA, 0.01 M NaCl, 1% SDS, pH 8.0), gently inverting the tube to mix thoroughly. The samples were treated with 32 μL of 6 M NaCl and proteinase K (150 μg mL^−1^) at 55 °C for 2 h, and lysozyme (10 mg mL^−1^) at room temperature. Next, 6 M NaCl was added to reach a final concentration of 1.5 M. The solution was chilled on ice for 10 min followed by centrifugation at 13,000 rpm for 3 min. The clear supernatant containing genomic DNA was transferred to another tube containing an equal volume of isopropanol. The tubes were inverted gently several times. The DNA was pelleted by centrifugation at 13,000 rpm for 3 min. The DNA pellet was then washed in 70% ethanol. The dried DNA pellet was resuspended in 100 μL of TE buffer (10 mM Tris–HCl, 1 mM EDTA, pH 8.0) and stored at 4 °C. DNA concentration was measured fluorometrically using the Qubit™ dsDNA HS Assay Kit (Thermo Fisher Scientific, Waltham, MA, USA).

The V3–V4 region of the 16S rRNA was amplified using the universal primers 341 F (5′‑CCTAYGGGRBGCASCAG‑3′) and 806R (5′‑GGACTACNNGGGTATCTAAT‑3′) (Klindworth et al. 2013). Amplification products were purified using AMPure XP magnetic beads (Beckman Coulter, Brea, CA, USA). Amplicon quality was assessed using the Agilent 5400 Fragment Analyzer System (Agilent Technologies, Santa Clara, CA, USA). The same amount of PCR products from each sample was pooled, end-repaired, A-tailed, and further ligated with Illumina adapters. The library was checked with Qubit and real-time PCR for quantification, while a bioanalyzer was used for size distribution detection. The libraries were then sequenced on the Illumina MiSeq platform (Illumina, San Diego, CA, USA) using 2 × 250 bp paired-end chemistry at the Ultrasequencing Service of Novogene Europe (Cambridge, United Kingdom).

All Illumina reads were analyzed using FastQC software (v0.11.4) (Andrews et al. 2010), and the Q30 score was maintained at a level of >92%. Further data processing included trimming, 16S rRNA analysis, and visualization using a workflow based on the DADA2 package of R software (Callahan et al. 2016) as described by Cerezo et al. (2024).

PICRUSt2 (v. 2.5, default settings, Douglas et al. 2019) was used to predict the functional metagenome content based on Amplicon Sequence Variant (ASV) abundances. Gene enrichment analysis was performed using KEGG pathways with the clusterProfiler R package (Yu et al. 2012), and enriched pathways were visualized using the pathview and ggplot2 R packages (Ginestet 2011; Luo and Brouwer 2013).

### RNA sequencing

Total RNA from intestinal samples was extracted following the TRIsure™ (Qiagen, Hilden, Germany) manufacturer’s instructions, and the eluted RNA was stored at −80 °C for subsequent analysis. Quantification and library sequencing of the total RNA were performed according to Cerezo et al. (2023).

Raw reads were processed to eliminate sequencing adapters, poly-N regions, and low-quality reads using fastqp software (Chen et al. 2018). Then, the Q20, Q30, and GC content of the clean data were computed, and all subsequent analyses were based on this clean, high-quality data.

The paired-end clean reads were mapped to the *D. rerio* reference transcriptome (Genome assembly GCF_000002035.6, obtained from https://www.ncbi.nlm.nih.gov/datasets/genome/GCF_000002035.6, accessed on May 9, 2017) using the Bowtie2 software (Langmead and Salzberg 2012). Transcript counts were generated using the sam2counts software (https://github.com/vsbuffalo/sam2counts/). KEGG pathway enrichment analysis was performed using the clusterProfiler R package (Yu et al. 2012). Enriched pathways were visualized using the ridgeplot function of the ggridges R package (Aldahmani et al. 2021).

### Statistical analysis

Statistical analyses were performed using GraphPad Prism (v.9.3.0.463) (Mavrevski et al. 2018) and R software. Prior to applying parametric tests, data normality and homoscedasticity were evaluated using the Shapiro–Wilk test and Levene’s test, respectively. Differences between the CTRL and VP groups were determined using Student’s* t*-test or the Mann–Whitney test when normality was not met. Differences in fish growth between dietary groups were assessed using a Student’s *t*-test.

For microbiota analysis, alpha diversity indices (Observed, Shannon, and Simpson) were compared between groups using Student’s *t*-tests. Beta diversity was evaluated through Principal Coordinate Analysis (PCoA) based on weighted UniFrac distances, and differences in microbial community composition were tested using permutational multivariate analysis of variance (PERMANOVA). Differences in classified taxa at the phylum and genus levels were analyzed by the Mann–Whitney test. Differential abundance of ASVs between dietary groups was determined using the DESeq2 package in R (Love et al. 2014), considering *p* < 0.05 as statistically significant.

Differences in predicted microbial functional pathways were computed using the ALDEx2 analysis tool (Gloor 2015), as recommended by the PICRUSt2 tutorial. Specifically, a Welch’s *t*-test with a Benjamini-Hochberg-corrected *p*-value of 0.05 was used to identify differentially abundant genes.

For transcriptomic analysis, differential gene expression was determined using the DESeq2 package in R, with transcripts having an absolute log_2_ fold change (|log_2_FC|) ≥ 1.3 and a false discovery rate (FDR) of <0.05 as significantly differentially expressed genes (DEGs). Statistical significance for KEGG pathway enrichment was determined using Benjamini–Hochberg correction, with an adjusted *p*-value threshold of ≤0.05.

## Results

No fish mortality was recorded during the experimental trial. Final body weight did not differ significantly between dietary treatments (final body weight (FBW) of 0.23 ± 0.02 g in the CTRL group and 0.24 ± 0.04 g in the VP group; Student’s* t*-test *p* > 0.05).

### Intestinal microbiota analysis

Clear sequences per sample of the 16S ribosomal RNA gene averaged 31,894,552 ± 2,465,167 in fish fed the CTRL diet and 31,357,227 ± 1,350,140 in fish fed the VP diet, and were clustered in 11,959 ASVs.

No significant differences were found between dietary groups for the assessed alpha diversity indices, namely Observed, Shannon, and Simpson indices (Student’s *t*-test *p* > 0.05; Table 2). Additionally, beta diversity did not cluster intestinal samples (Supplemental Fig. S1), and no significant alterations in the intestinal microbiota were highlighted by the PERMANOVA test (*p* > 0.05).

**Table 2:** Alpha diversity indices of the gut microbiota in zebrafish fed experimental diets

	CTRL	VP	*p*-value
Observed	333.8 ± 103.7	336.4 ± 130.2	0.965
Shannon index	3.4 ± 0.5	3.4 ± 0.5	0.894
Simpson index	0.91 ± 0.1	0.91 ± 0.1	0.752

**Fig. 1:**
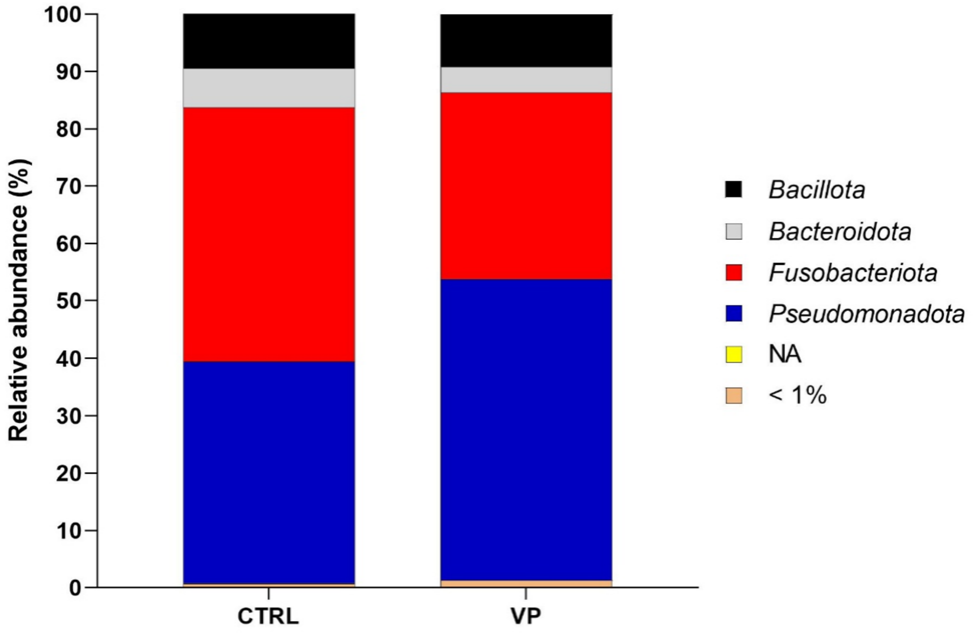
Mean relative abundance (percentage) at the phylum level in the gut microbiota of zebrafish fed the experimental diets. <1% indicates a mean relative abundance of less than 1%. NA represents unassigned taxa

After filtering ASVs whose abundance was lower than 10 in at least 10% of samples, 501 ASVs were used for taxonomic analyses. *Pseudomonadota* and *Fusobacteriota*, followed by *Bacillota* and *Bacteroidota*, were the predominant phyla detected in the intestines of zebrafish fed with both diets (Fig. 1, Supplemental Fig. S2). No significant differences were observed between treatments at the phylum level (Mann–Whitney test, *p* > 0.05).

**Fig. 2:**
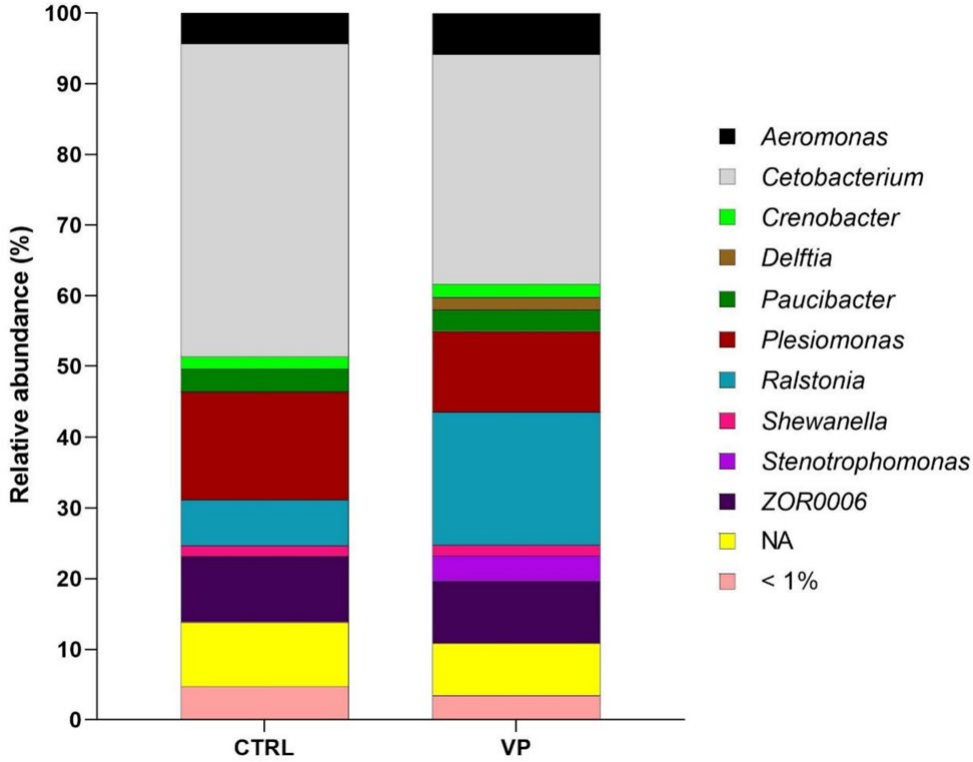
Mean relative abundance (percentage) at the genus level of gut microbiota of zebrafish fed the experimental diets. <1% indicates a mean relative abundance of less than 1%. NA represents unassigned taxa

At the genus level, fish fed the CTRL diet were dominated by *Cetobacterium* (44.3%) and *Plesiomonas* (15.2%) (Fig. 2; Supplemental Fig. S3). In contrast, fish fed the VP diet showed a reduced relative abundance of *Cetobacterium* (32.5%) and *Plesiomonas* (11.4%), together with an increased abundance of *Ralstonia* (18.9% in the VP diet vs. 6.5% in the CTRL diet). Additionally, *Stenotrophomonas* (3.5%) and *Delftia* (1.8%) were detected exclusively in fish fed the VP diet and were absent in the CTRL group. However, none of the observed changes at the genus level were statistically significant (Mann–Whitney test, *p* ≥ 0.05).

As the analyses conducted at the phylum and genus levels did not identify significant differences between treatments, a more detailed assessment was carried out at the ASV level.

The DESeq2 algorithm detected significant differences in ASV abundances between dietary groups (*p* < 0.05; Supplemental Table S1). Different ASVs affiliated with the genus *Cetobacterium* showed increased abundance in both dietary treatments. The VP group exhibited a significant augmentation in the abundance of ASVs related to the genera *Aeromonas*, *Crenobacter*, *Delftia*, *Plesiomonas, Ralstonia*, *Shewanella, Stenotrophomonas*, and *ZOR0006* compared to the CTRL group (Supplemental Table S1).

To determine whether these taxonomic shifts at the ASV level were associated with changes in the predicted functional potential of the microbiota, microbial metabolic pathways were inferred using PICRUSt2 and statistically evaluated using the ALDEx2 tool. Despite differences in ASV abundances, no statistically significant differences in microbial functions were observed between groups (Supplemental Fig. S4).

### RNA sequencing

The RNA sequencing final read count averaged 86,508,702 ± 3,880,314 per individual in fish fed the CTRL diet (range: 79,933,684–93,549,640) and 90,101,947 ± 10,003,259 in fish fed the VP diet (range: 81,856,584–114,176,584). The overall mapping rate was 57.6 ± 6.2% for the CTRL group and 61.8 ± 6.8% for the VP group.

**Fig. 3:**
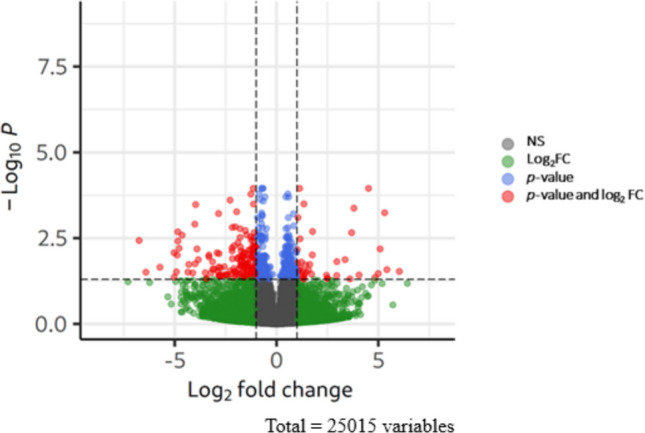
Volcano plot showing DEGs in the gut of zebrafish fed the experimental diets. NS (gray dots) represent non-significant differences. Log2FC (green dots) indicates values where log2FC < 1.3. *p*-value (blue dots) represents data points that are significant at the *p* < 0.05 threshold. Red dots represent DEGs that have a *p*-value and Log2FC above ≥|1.3|

Out of a total of 25,015 genes identified, 201 were differentially expressed (DEGs) in the two groups, as confirmed by DESeq2 analysis (Log2 Fold Change absolute value > 1.3; *p* < 0.05) (Fig. 3). Of these, 161 genes were downregulated, and 40 genes were upregulated in fish fed the VP diet compared to the CTRL group (Supplemental Table S2).

The identification of differentially expressed genes was conducted using over-representation analyses (ORA) of Gene Ontology (GO) Biological Processes and KEGG pathways. These analyses showed upregulation of metabolites associated with lipid transport and metabolism, regulation of glucose transport, and fibroblast proliferation in the intestine, as well as downregulation of immune‑related pathway responses (Supplemental Fig. S5–S7). Non-significant differences were shown in KEGG ORA results (*p* > 0.05).

Although ORA provided an initial overview of functional categories associated with the differentially expressed genes, this approach is inherently limited to DEGs and may overlook subtle but coordinated shifts across functionally related gene sets (Stead et al. 2025). Given that the VP diet elicited modest yet concerted transcriptional changes distributed across many genes, we applied Gene Set Enrichment Analysis (GSEA), which evaluates all genes ranked by fold change and is therefore more sensitive to detecting pathway-level regulation under this condition.

**Fig. 4:**
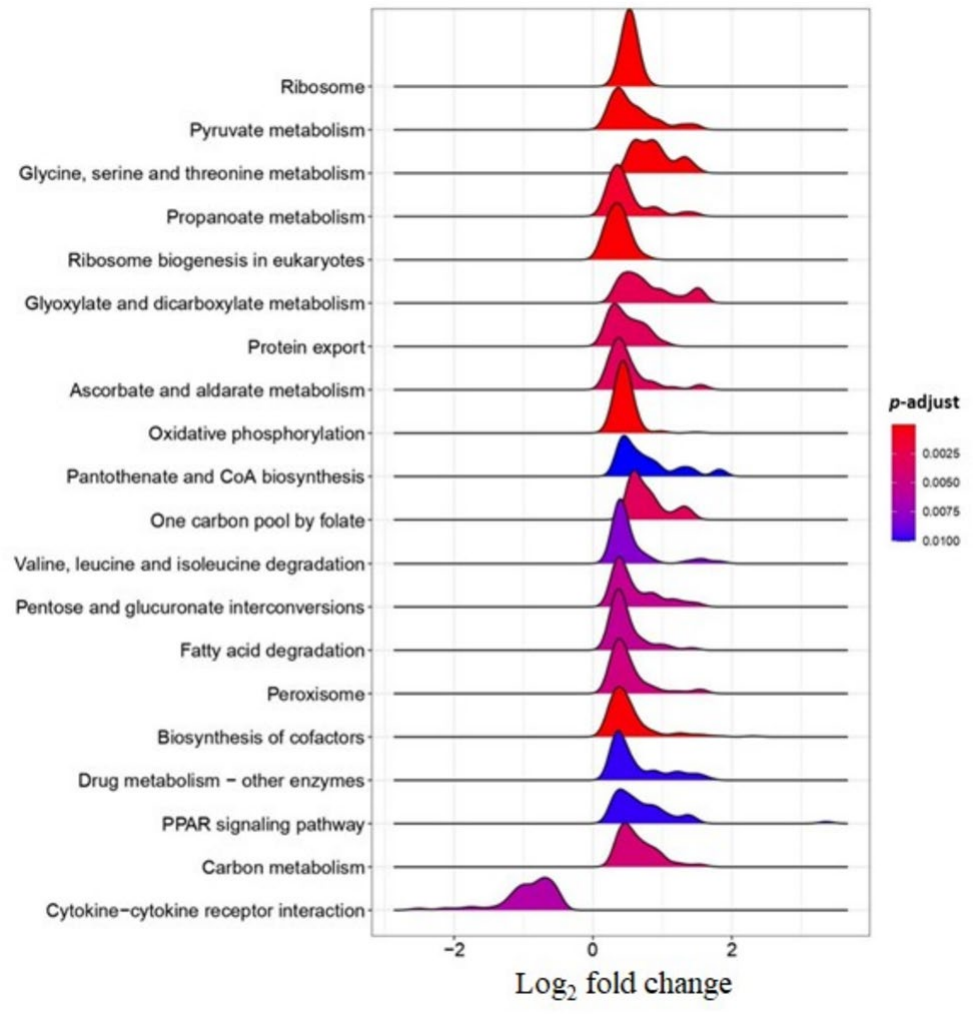
Ridgeplot of KEGG pathways by GSEA analysis. Negative values represent downregulated pathways, whereas positive values represent upregulated pathways in response to the dietary administration of *V. proteolyticus* DCF12.2

GSEA indicated that fish fed the VP diet exhibited downregulation of the cytokine-cytokine receptor interaction pathway compared to fish fed the CTRL diet (Fig. 4). Conversely, several metabolic pathways were overexpressed in fish fed the VP diet, including those involved in lipid metabolism (fatty acid degradation), amino acid metabolism (propanoate metabolism; valine, leucine, and isoleucine degradation; glycine, serine, and threonine metabolism; one-carbon pool by folate), cofactor biosynthesis and detoxification (pantothenate and CoA biosynthesis; biosynthesis of cofactors; drug metabolism—other enzymes), carbohydrate metabolism (pentose and glucuronate interconversion; ascorbate and aldarate metabolism; carbon metabolism), energy metabolism (oxidative phosphorylation) and protein processing (ribosome biogenesis in eukaryotes; ribosome; protein export; peroxisome; PPAR (peroxisome proliferator–activated receptors) signaling pathway).

## Discussion

The microorganisms that inhabit the intestinal tract play a significant role in promoting digestion, absorption, nutrient metabolism, and immunity in aquatic animals (Nayak 2010). Studies have shown that disruptions in gut microbial homeostasis can induce the growth of potential pathogenic bacteria (Hao et al. 2021). In our study, supplementation of inactivated *V. proteolyticus* DCF12.2 into a practical zebrafish diet did not induce significant changes in alpha or beta diversity of the intestinal microbiota. These findings suggest that, under the tested conditions, the intestinal microbiota maintained a stable community structure despite the introduction of the *V. proteolyticus* DCF12.2 postbiotic, consistent with reports for other postbiotics (Mohapatra et al. 2012; Rocha et al. 2023).

*Pseudomonadota* and *Fusobacteriota* were the dominant phyla in both groups, consistent with previous reports on the intestinal microbiota of zebrafish (Roeselers et al. 2011; López Nadal et al. 2020; Cornualt et al. 2022). The most abundant genus in the CTRL group was *Cetobacterium,* which has been frequently identified as a dominant taxon in the intestinal microbiota of zebrafish and other freshwater fish species (Garibay-Valdez et al. 2024). *Cetobacterium* is recognized for its role in vitamin B12 synthesis and carbohydrate metabolism, which contributes to enhancing zebrafish performance (Wang et al. 2021; Li et al. 2023; Qi et al. 2023).

In the present study, although no significant differences in *Cetobacterium* genus abundance were observed, DESeq2 analysis identified significant variations in ASVs assigned to *Cetobacterium* in both groups. Furthermore, several ASVs assigned to *Aeromonas*, *Crenobacter*, *Delftia*, *Plesiomonas*, *Ralstonia*, *Shewanella*, *Stenotrophomonas*, and *ZOR0006* were significantly more represented in the VP group. Although certain *Aeromonas* strains are associated with pathogenic infections (Fernández-Bravo and Figueras 2020), they are also commonly found in the intestines of freshwater fish, where they can play commensal roles (Nayak 2010). Conversely, *Crenobacter* species possess an extensive enzymatic repertoire involved in carbohydrate and lipid metabolism, reflecting an adaptive metabolic profile suited to environments rich in diverse organic substrates (Zhang et al. 2024). This suggests potential roles in nutrient turnover within host-associated and aquatic microbial communities (Shi et al. 2021). *Delftia* species are recognized for their ability to degrade toxins and aromatic compounds (Vásquez-Piñeros et al. 2018), and their presence was associated with improved intestinal health and structural integrity in juvenile black sea bream (*Acanthopagrus schlegelii*) (Sagada et al. 2025). *Plesiomonas* is a common intestinal taxon that exhibits both commensal and opportunistic behavior depending on the host’s physiological status and environmental context (Siriyappagouder et al. 2018; Zhong et al. 2022; Garibay-Valdez et al. 2024). Members of the genus *Ralstonia* are known to display antimicrobial activity and to produce secondary metabolites beneficial to the host (Jami et al. 2015). This genus has been reported in several fish species, including European sea bass (*Dicentrarchus labrax*) (Carda-Diéguez et al. 2014), gilthead seabream (*Sparus aurata*) (Cerezo-Ortega et al. 2021), yellowtail kingfish (*Seriola lalandi*) (Dam et al. 2020), and turbot (*Scophthalmus maximus*) (Wei et al. 2023). *Stenotrophomonas* species inhabit diverse ecological niches and are associated with non-pathogenic traits such as chitin degradation and antifungal activity (Brzezinska et al. 2014; Rosado et al. 2019), although some strains have been identified as opportunistic pathogens in fish (Brooke 2012; Abraham and Adikesavalu 2016). *Shewanella* is another metabolically versatile genus frequently found in aquatic environments, with members capable of participating in nutrient cycling and redox balance within microbial communities (Dailey et al. 2015). Several studies have also reported beneficial effects of dietary *Shewanella* strains on fish health (Cámara-Ruiz et al. 2020).

The limited read length of the 16S ribosomal RNA amplicon sequencing for the V3-V4 amplicon, combined with the inherent low taxonomic resolution of the conserved 16S rRNA gene, hinders species-level assignments (Martínez-Porchas et al. 2016; Commichaux et al. 2024). However, studies have emphasized that even minor ASV-level variations can play crucial roles in maintaining microbiome function and overall host health (Legrand et al. 2018; Sadeghi et al. 2023). Our results, however, showed that despite compositional changes at the ASV level, no significant differences were observed in predicted microbial functions between treatments. This functional stability suggests that the subtle alterations detected among ASVs may perform similar metabolic roles, thereby maintaining ecological balance and overall gut homeostasis (Cerezo et al. 2024).

The recent application of comparative pathway analysis techniques, particularly threshold-free pathway analysis such as Gene Set Enrichment Analysis (GSEA), has provided valuable insights into the dysregulation of fundamental biological pathways in mammals (Riddell and Crewther 2017).

In our study, GSEA detected significant transcriptomic alterations in zebrafish intestines following dietary inclusion of *V. proteolyticus* DCF12.2, suggesting impacts on immune regulation, nutrient metabolism, and cellular homeostasis. The VP diet induced upregulation of metabolic pathways involved in nutrient utilization and cellular metabolism. In this sense, the activation of amino acid metabolism pathways, including those for valine, leucine, and isoleucine degradation, as well as glycine, serine, and threonine metabolism, suggests an increased capacity for protein turnover, biosynthesis, and energy generation (Li et al. 2021). Since these pathways play crucial roles in cell proliferation, tissue repair, and antioxidant defense, *V. proteolyticus* DCF12.2 may have contributed to improved metabolic activity and maintenance of gut function rather than directly promoting somatic growth.

The overexpression of oxidative phosphorylation and peroxisomal pathways in fish fed the VP diet further suggests improved mitochondrial function and redox homeostasis. Enhanced mitochondrial activity is crucial for maintaining intestinal epithelial integrity, while peroxisomal pathways play a key role in detoxification and lipid metabolism, thereby preventing oxidative damage (Rath et al. 2018; Guerbette et al. 2022). Moreover, the upregulation of ribosome biogenesis and protein export pathways indicates a potential enhancement in protein synthesis and cellular repair mechanisms, which might be beneficial for maintaining gut health (De Saedeleer et al. 2021; Jiao et al. 2023). However, as no differences in growth were observed, the activated pathways may have supported tissue maintenance, epithelial renewal, and metabolic adaptation, with the derived energy preferentially allocated to homeostatic processes rather than biomass accretion.

Furthermore, the VP diet induced an upregulation of pathways related to lipid metabolism, particularly fatty acid degradation. This finding is complex to interpret. On one hand, enhanced lipid catabolism could suggest improved energy mobilization and metabolic efficiency, which may be favorable for intestinal health and support tissue maintenance (Guo et al. 2019; Xie et al. 2022). However, on the other hand, increased lipid metabolism, particularly when not balanced by corresponding changes in growth or energy demand, has been associated with the development of low-grade chronic inflammation, insulin resistance, or ectopic lipid deposition in some vertebrate models (Zhang et al. 2018; Andersen 2022). Such outcomes may lead to cellular stress or metabolic dysregulation over time (Vaziri 2014; Lee and Olefsky 2021).

One of the most notable findings was the downregulation of the cytokine–cytokine receptor interaction pathway in fish fed the VP diet. This pathway plays a central role in immune surveillance and orchestrating inflammatory responses (Soliman and Barreda 2023). A decrease in transcription of genes associated with this pathway may reduce the availability of cytokine receptors on the surface of immune cells, potentially weakening their capacity to sense and respond to immune stimuli (Zou and Secombes 2016). While this could reflect a controlled inflammatory tone under homeostatic conditions, it also raises concerns about possible immunosuppression, as a reduced capacity to transduce cytokine signals may impair the host’s ability to mount effective responses against pathogens or resolve inflammation efficiently (O’Neill 2006; Mogensen 2009).

Taken together, the concurrent downregulation of cytokine receptor signalling and upregulation of lipid metabolism might reflect an altered immunometabolic state, in which reduced immune sensitivity coexists with heightened lipid turnover. This dual modulation suggests the possibility of a disrupted feedback loop between the immune and metabolic systems, potentially leading to a state of subclinical dysfunction (Park et al. 2025). Such interplay between immune suppression and metabolic activation warrants further investigation, particularly under challenging conditions or prolonged feeding periods. Overall, the observed transcriptomic modulations did not translate into short-term somatic growth, as no significant differences in growth performance were detected between dietary groups, supporting the interpretation that the intestinal response was primarily adaptive and functional rather than growth-promoting. Further studies should aim to validate key differentially expressed genes using targeted approaches, such as qPCR, to strengthen the robustness of the transcriptomic findings. Additionally, extended feeding trials would be valuable for determining whether the observed metabolic and transcriptional modulation ultimately translates into measurable improvements in growth performance, as molecular responses may precede detectable phenotypic changes.

## Conclusion

Our results indicate that dietary supplementation with *V. proteolyticus* DCF12.2 postbiotics modulates the functional state of zebrafish intestinal cells, characterized by a reduction in immune-related signaling alongside the upregulation of metabolic pathways associated with nutrient utilization and energy production. This response suggests a coordinated metabolic adjustment to postbiotic supplementation at the host level. Notably, these effects occurred without affecting growth performance or inducing major shifts in the overall intestinal microbiota composition.

Collectively, these findings provide new evidence that postbiotics derived from *V. proteolyticus* can exert targeted effects on host intestinal physiology independently of large-scale microbiota restructuring, supporting their potential as functional ingredients in aquafeeds.

## Supplementary Information

Below is the link to the electronic supplementary material.

ESM 1 DOCX (1.02 MB)

## Data Availability

The datasets generated and analyzed in this study are available in the NCBI repository ([https://www.ncbi.nlm.nih.gov/](https://www.ncbi.nlm.nih.gov)). Microbiota data are deposited under BioProject accession number PRJNA1380208 (BioSamples SAMN53854348–SAMN53854365), and RNA-seq data under BioProject accession number PRJNA1379280 (BioSamples SAMN53858022–SAMN53858043).

## References

[CR1] Abd El-Hack ME, El-Saadony MT, Shafi ME, Qattan SYA, Batiha GE, Khafaga AF, Abdel-Moneim AME, Alagawany M (2020) Probiotics in poultry feed: a comprehensive review. J Anim Physiol Anim Nutr 104(6):1835–1850. 10.1111/JPN.13454 10.1111/jpn.13454 32996177

[CR2] Abraham TJ, Adikesavalu H (2016) Association of Stenotrophomonas maltophilia in African catfish, Clarias gariepinus (Burchell, 1822) fry mortalities with dropsy. Int J Aquac 6(13):1–5. 10.5376/ija.2016.06.0013

[CR3] Aldahmani S, Zoubeidi T, Aldahmani MS (2021) Package ‘GGRidge’. See https://CRAN.R-project.org/package=ggridges

[CR4] Aleström P, D’Angelo L, Midtlyng PJ, Schorderet DF, Schulte-Merker S, Sohm F, Warner S (2020) Zebrafish: housing and husbandry recommendations. Lab Anim 54(3):213–224. 10.1177/0023677219869037 31510859 10.1177/0023677219869037 PMC7301644

[CR5] Andersen CJ (2022) Lipid metabolism in inflammation and immune function. Nutrients 14(7):1414. 10.3390/NU14071414 35406026 10.3390/nu14071414 PMC9002396

[CR6] Andrews S, Krueger F, Segonds-Pichon A, Biggins L, Krueger C, Wingett S (2010) FastQC. A quality control tool for high throughput sequence data, 370. www.bioinformatics.babraham.ac.uk/projects/fastqc/.

[CR7] Brooke JS (2012) Stenotrophomonas maltophilia: an emerging global opportunistic pathogen. Clin Microbiol Rev 25(1):2–41. 10.1128/cmr.00019-11 22232370 10.1128/CMR.00019-11 PMC3255966

[CR8] Brzezinska MS, Jankiewicz U, Burkowska A, Walczak M (2014) Chitinolytic microorganisms and their possible application in environmental protection. Curr Microbiol 68(1):71–81. 10.1007/S00284-013-0440-4/TABLES/2 23989799 10.1007/s00284-013-0440-4 PMC3889922

[CR9] Callahan BJ, McMurdie PJ, Rosen MJ, Han AW, Johnson AJA, Holmes SP (2016) DADA2: high-resolution sample inference from Illumina amplicon data. Nat Methods 13(7):581–583. 10.1038/nmeth.3869 27214047 10.1038/nmeth.3869 PMC4927377

[CR10] Cámara-Ruiz M, Balebona MC, Moriñigo MÁ, Esteban MÁ (2020) Probiotic *Shewanella putrefaciens* (SpPdp11) as a fish health modulator: a review. Microorganisms 8(12):1990. 10.3390/microorganisms8121990 33327443 10.3390/microorganisms8121990 PMC7764857

[CR11] Carda-Diéguez M, Mira A, Fouz B (2014) Pyrosequencing survey of intestinal microbiota diversity in cultured sea bass (*Dicentrarchus labrax*) fed functional diets. FEMS Microbiol Ecol 87(2):451–459. 10.1111/1574-6941.12236 24730648 10.1111/1574-6941.12236

[CR12] Cerezo IM, Pérez-Gómez O, Bautista R, Seoane P, Esteban MÁ, Balebona MC, Moriñigo MA, Tapia-Paniagua ST (2023) SpPdp11 administration in diet modified the transcriptomic response and its microbiota associated in mechanically induced wound *Sparus aurata* skin. Animals 13(2):193. 10.3390/ANI13020193 36670734 10.3390/ani13020193 PMC9854838

[CR13] Cerezo IM, Herrada EA, Fernández-Gracia J, Sáez-Casado MI, Martos-Sitcha JA, Moriñigo MA, Tapia-Paniagua ST (2024) Analyzing bacterial networks and interactions in skin and gills of *Sparus aurata* with microalgae-based additive feeding. Sci Rep 14(1):1–14. 10.1038/S41598-024-81822-Z 39738183 10.1038/s41598-024-81822-z PMC11686111

[CR14] Cerezo-Ortega IM, Di Zeo-Sánchez DE, García-Márquez J, Ruiz-Jarabo I, Sáez-Casado MI, Balebona MC, Moriñigo MA, Tapia-Paniagua ST (2021) Microbiota composition and intestinal integrity remain unaltered after the inclusion of hydrolysed *Nannochloropsis gaditana* in *Sparus aurata* diet. Sci Rep 11(1):1–16. 10.1038/s41598-021-98087-5 34548549 10.1038/s41598-021-98087-5 PMC8455595

[CR15] Charlon N, Bergot P (1984) Rearing system for feeding fish larvae on dry diets. Trial with carp (*Cyprinus carpio* L.) larvae. Aquaculture 41:1–9. 10.1016/0044-8486(84)90384-3

[CR16] Chen S, Zhou Y, Chen Y, Gu J (2018) fastp: an ultra-fast all-in-one FASTQ preprocessor. Bioinformatics 34(17):i884–i890. 10.1093/BIOINFORMATICS/BTY560 30423086 10.1093/bioinformatics/bty560 PMC6129281

[CR17] Choudhury TG, Kamilya D (2019) Paraprobiotics: an aquaculture perspective. Rev Aquac 11(4):1258–1270. 10.1111/RAQ.12290

[CR18] Commichaux S, Luan T, Muralidharan HS, Pop M (2024) Database size positively correlates with the loss of species-level taxonomic resolution for the 16S rRNA and other prokaryotic marker genes. PLoS Comput Biol 20(8):e1012343. 10.1371/journal.pcbi.1012343 39102435 10.1371/journal.pcbi.1012343 PMC11326629

[CR19] Cornuault JK, Byatt G, Paquet ME, De Koninck P, Moineau S (2022) Zebrafish: a big fish in the study of the gut microbiota. Curr Opin Biotechnol 73:308–313. 10.1016/J.COPBIO.2021.09.007 34653834 10.1016/j.copbio.2021.09.007

[CR20] Dailey FE, McGraw JE, Jensen BJ, Bishop SS, Lokken JP, Dorff KJ, Ripley MP, Munro JB (2015) The microbiota of freshwater fish and freshwater niches contain omega-3 fatty acid-producing Shewanella species. Appl Environ Microbiol 82(1):218–231. 10.1128/AEM.02266-15 26497452 10.1128/AEM.02266-15 PMC4702627

[CR21] Dam CTM, Booth M, Pirozzi I, Salini M, Smullen R, Ventura T, Elizur A (2020) Alternative feed raw materials modulate intestinal microbiota and its relationship with digestibility in yellowtail kingfish *Seriola lalandi*. Fishes 5(2):14. 10.3390/fishes5020014

[CR22] de Almada CN, Almada CN, Martinez RCR, Sant’Ana AS (2016) Paraprobiotics: evidences on their ability to modify biological responses, inactivation methods and perspectives on their application in foods. Trends Food Sci Technol 58:96–114. 10.1016/j.tifs.2016.09.011

[CR23] De Saedeleer B, Malabirade A, Ramiro-Garcia J, Habier J, Trezzi J-P, Peters SL, Daujeumont A, Halder R, Jäger C, Busi SB, May P, Oertel W, Mollenhauer B, Laczny CC, Hettich RL, Wilmes P (2021) Systematic characterization of human gut microbiome-secreted molecules by integrated multi-omics. ISME Commun 1(1):82. 10.1038/S43705-021-00078-0 35106519 10.1038/s43705-021-00078-0 PMC7612290

[CR24] Douglas GM, Maffei VJ, Zaneveld J, Yurgel SN, Brown JR, Taylor CM, Huttenhower C, Langille MG (2019) PICRUSt2: an improved and extensible approach for metagenome inference. BioRxiv. 10.1101/672295

[CR25] Fernández-Bravo A, Figueras MJ (2020) An update on the genus *Aeromonas*: taxonomy, epidemiology, and pathogenicity. Microorganisms 8(1):129. 10.3390/microorganisms8010129 31963469 10.3390/microorganisms8010129 PMC7022790

[CR26] García-Márquez J, Vizcaíno AJ, Barany A, Galafat A, Acién G, Figueroa FL, Alarcón FJ, Mancera JM, Martos-Sitcha JA, Arijo S, Abdala-Díaz RT (2023a) Evaluation of the combined administration of *Chlorella fusca* and *Vibrio proteolyticus* in diets for *Chelon labrosus*: effects on growth, metabolism, and digestive functionality. Animals 13(4):589. 10.3390/ANI13040589/S1 36830376 10.3390/ani13040589 PMC9951767

[CR27] García-Márquez J, Álvarez-Torres D, Cerezo IM, Domínguez-Maqueda M, Figueroa FL, Alarcón FJ, Acién G, Martínez-Manzanares E, Abdala-Díaz RT, Béjar J, Arijo S (2023b) Combined dietary administration of *Chlorella fusca* and ethanol-inactivated *Vibrio proteolyticus* modulates intestinal microbiota and gene expression in *Chelon labrosus*. Animals 13(21):3325. 10.3390/ANI13213325/S1 37958080 10.3390/ani13213325 PMC10648860

[CR28] Garibay-Valdez E, Olivas-Bernal CA, Vargas-Albores F, Martínez-Porchas M, García-Godínez DM, Medina-Félix D, Martínez-Córdova LR, Cicala F (2024) Deciphering the gut microbiota of zebrafish, the most used fish as a biological model: a meta-analytic approach. Comp Biochem Physiol A Mol Integr Physiol 297:111713. 10.1016/J.CBPA.2024.111713 39074543 10.1016/j.cbpa.2024.111713

[CR29] Ginestet C (2011) ggplot2: elegant graphics for data analysis. J R Stat Soc Ser A Stat Soc 174(1):245–246. 10.1111/j.1467-985X.2010.00676_9.x

[CR30] Giri SS, Sen SS, Chi C, Kim HJ, Yun S, Park SC, Sukumaran V (2015) Effect of cellular products of potential probiotic bacteria on the immune response of *Labeo rohita* and susceptibility to *Aeromonas hydrophila* infection. Fish Shellfish Immunol 46(2):716–722. 10.1016/J.FSI.2015.08.012 26282681 10.1016/j.fsi.2015.08.012

[CR31] Giri SS, Jun JW, Yun S, Kim HJ, Kim SG, Kim SW, Woo KJ, Han SJ, Oh WT, Kwon J, Sukumaran V, Park SC (2020) Effects of dietary heat-killed *Pseudomonas aeruginosa* strain VSG2 on immune functions, antioxidant efficacy, and disease resistance in *Cyprinus carpio*. Aquaculture 514:734489. 10.1016/J.AQUACULTURE.2019.734489

[CR32] Gloor G (2015) ALDEx2: ANOVA-like differential expression tool for compositional data. ALDEX Manual Modular 20:1–11

[CR33] Guerbette T, Boudry G, Lan A (2022) Mitochondrial function in intestinal epithelium homeostasis and modulation in diet-induced obesity. Mol Metab 63:101546. 10.1016/J.MOLMET.2022.101546 35817394 10.1016/j.molmet.2022.101546 PMC9305624

[CR34] Guo J, Zhou Y, Zhao H, Chen WY, Chen YJ, Lin SM (2019) Effect of dietary lipid level on growth, lipid metabolism and oxidative status of largemouth bass, *Micropterus salmoides*. Aquaculture 506:394–400. 10.1016/J.AQUACULTURE.2019.04.007

[CR35] Hammer HS (2020) Water quality for zebrafish culture. In: Cartner SC, Eisen JS, Farmer SF, Guillemin KJ, Kent ML, Sanders GE (eds) The zebrafish in biomedical research: biology, husbandry, diseases, and research applications. Academic Press, Elsevier, London, UK, p pp 321-335

[CR36] Hao Q, Teame T, Wu X, Ding Q, Ran C, Yang Y, Xing Y, Zhang Z, Zhou Z (2021) Influence of diet shift from bloodwormto formulated feed on growth performance, gut microbiota structure and function in early juvenile stages of hybridsturgeon (*Acipenser baerii × Acipenser schrenckii*). Aquac 533:736165. 10.1016/j.aquaculture.2020.736165

[CR37] Hill C, Guarner F, Reid G, Gibson GR, Merenstein DJ, Pot B, Morelli L, Canani RB, Flint HJ, Salminen S, Calder PC, Sanders ME (2014) Expert consensus document: the international scientific association for probiotics and prebiotics consensus statement on the scope and appropriate use of the term probiotic. Nat Rev Gastroenterol Hepatol 11(8):506–514. 10.1038/NRGASTRO.2014.66 24912386 10.1038/nrgastro.2014.66

[CR38] Jami M, Ghanbari M, Kneifel W, Domig KJ (2015) Phylogenetic diversity and biological activity of culturable *Actinobacteria* isolated from freshwater fish gut microbiota. Microbiol Res 175:6–15. 10.1016/J.MICRES.2015.01.009 25662514 10.1016/j.micres.2015.01.009

[CR39] Jiao L, Liu Y, Yu XY, Pan X, Zhang Y, Tu J, Song YH, Li Y (2023) Ribosome biogenesis in disease: new players and therapeutic targets. Signal Transduct Target Ther 8(1):1–22. 10.1038/s41392-022-01285-4 36617563 10.1038/s41392-022-01285-4 PMC9826790

[CR40] Kerry RG, Patra JK, Gouda S, Park Y, Shin HS, Das G (2018) Benefaction of probiotics for human health: a review. J Food Drug Anal 26(3):927–939. 10.1016/J.JFDA.2018.01.002 29976412 10.1016/j.jfda.2018.01.002 PMC9303019

[CR41] Klindworth A, Pruesse E, Schweer T, Peplies J, Quast C, Horn M, Glöckner FO (2013) Evaluation of general 16S ribosomal RNA gene PCR primers for classical and next-generation sequencing-based diversity studies. Nucleic Acids Res 41(1):e1–e1. 10.1093/NAR/GKS808 22933715 10.1093/nar/gks808 PMC3592464

[CR42] Langmead B, Salzberg SL (2012) Fast gapped-read alignment with Bowtie 2. Nat Methods 9(4):357–359. 10.1038/NMETH.1923 22388286 10.1038/nmeth.1923 PMC3322381

[CR43] Lee YS, Olefsky J (2021) Chronic tissue inflammation and metabolic disease. Genes Dev 35(5–6):307–328. 10.1101/GAD.346312.120 33649162 10.1101/gad.346312.120 PMC7919414

[CR44] Legrand TPRA, Catalano SR, Wos-Oxley ML, Stephens F, Landos M, Bansemer MS, Stone DAJ, Qin JG, Oxley APA (2018) The inner workings of the outer surface: skin and gill microbiota as indicators of changing gut health in yellowtail kingfish. Front Microbiol 8:2664. 10.3389/fmicb.2017.02664 29379473 10.3389/fmicb.2017.02664 PMC5775239

[CR45] Li X, Zheng S, Wu G (2021) Nutrition and functions of amino acids in fish. In: Wu G (eds) Amino acids in nutrition and health. Adv Exp Med Biol 1285:133–168. Springer, Cham. 10.1007/978-3-030-54462-1_8 10.1007/978-3-030-54462-1_8 33770406

[CR46] Li S, Yang H, Jin Y, Hao Q, Liu S, Ding Q, Yao Y, Yang Y, Ran C, Wu C, Li S, Cheng K, Hu J, Liu H, Zhang Z, Zhou Z (2023) Dietary cultured supernatant mixture of *Cetobacterium somerae* and *Lactococcus lactis* improved liver and gut health, and gut microbiota homeostasis of zebrafish fed with high-fat diet. Fish Shellfish Immunol 142:109139. 10.1016/J.FSI.2023.109139 37821002 10.1016/j.fsi.2023.109139

[CR47] López Nadal A, Ikeda-Ohtsubo W, Sipkema D, Peggs D, McGurk C, Forlenza M, Wiegertjes GF, Brugman S (2020) Feed, microbiota, and gut immunity: using the zebrafish model to understand fish health. Front Immunol 11:512428. 10.3389/FIMMU.2020.00114/EPUB 10.3389/fimmu.2020.00114 PMC7014991 32117265

[CR48] Love MI, Huber W, Anders S (2014) Moderated estimation of fold change and dispersion for RNA-seq data with DESeq2. Genome Biol 15:550. 10.1186/s13059-014-0550-8 25516281 10.1186/s13059-014-0550-8 PMC4302049

[CR49] Luo W, Brouwer C (2013) Pathview: an R/Bioconductor package for pathway-based data integration and visualization. Bioinformatics 29(14):1830–1831. 10.1093/bioinformatics/btt285 23740750 10.1093/bioinformatics/btt285 PMC3702256

[CR50] Martínez G, Shaw EM, Carrillo M, Zanuy S (1998) Protein salting-out method applied to genomic DNA isolation from fish whole blood. Biotechniques 24(2):238–239. 10.2144/98242BM14 9494722 10.2144/98242bm14

[CR51] Martínez-Porchas M, Villalpando-Canchola E, Vargas-Albores F (2016) Significant loss of sensitivity and specificity in the taxonomic classification occurs when short 16S rRNA gene sequences are used. Heliyon, 2(9). 10.1016/j.heliyon.2016.e00170 10.1016/j.heliyon.2016.e00170 PMC5037269 27699286

[CR52] Mavrevski R, Traykov M, Trenchev I, Trencheva M (2018) Approaches to modeling of biological experimental data with GraphPad Prism software. WSEAS Trans Syst Control 13:242–247

[CR53] Medina A, Moriñigo MÁ, Arijo S (2020) Selection of putative probiotics based on antigen-antibody cross-reaction with *Photobacterium damselae* subsp. *piscicida* and *Vibrio harveyi* for use in Senegalese sole (*Solea senegalensis*). Aquac Rep 17:100366. 10.1016/J.AQREP.2020.100366

[CR54] Medina A, García-Márquez J, Moriñigo MÁ, Arijo S (2023) Effect of the potential probiotic *Vibrio proteolyticus* DCF12.2 on the immune system of *Solea senegalensis* and protection against *Photobacterium damselae* subsp. *piscicida* and *Vibrio harveyi*. Fishes 8(7):344. 10.3390/FISHES8070344

[CR55] Mogensen TH (2009) Pathogen recognition and inflammatory signaling in innate immune defenses. Clin Microbiol Rev 22(2):240–273. 10.1128/CMR.00046-08/19366914 10.1128/CMR.00046-08 PMC2668232

[CR56] Mohapatra S, Chakraborty T, Prusty AK, Das P, Paniprasad K, Mohanta KN (2012) Use of different microbial probiotics in the diet of rohu, *Labeo rohita* fingerlings: effects on growth, nutrient digestibility and retention, digestive enzyme activities and intestinal microflora. Aquac Nutr 18(1):1–11. 10.1111/j.1365-2095.2011.00866.x

[CR57] Nayak SK (2010) Role of gastrointestinal microbiota in fish. Aquac Res 41(11):1553–1573. 10.1111/j.1365-2109.2010.02546.x

[CR58] O’Neill LAJ (2006) Targeting signal transduction as a strategy to treat inflammatory diseases. Nat Rev Drug Discov 5(7):549–563. 10.1038/NRD2070 16773072 10.1038/nrd2070

[CR59] Park J, Lee Y, Lee JY, Kang HY, Kim S, Kim S, Kim BS, Kim DH (2025) Overfeeding in rainbow trout (*Oncorhynchus mykiss*): Metabolic disruptions, impaired immunity, and increased infection risk. Fish Shellfish Immunol 160:110224. 10.1016/j.fsi.2025.110224 39988219 10.1016/j.fsi.2025.110224

[CR60] Qi X, Zhang Y, Zhang Y, Luo F, Song K, Wang G, Ling F (2023) Vitamin B12 produced by *Cetobacterium somerae* improves host resistance against pathogen infection through strengthening the interactions within gut microbiota. Microbiome 11(1):1–25. 10.1186/S40168-023-01574-2 37322528 10.1186/s40168-023-01574-2 PMC10268390

[CR61] Rath E, Moschetta A, Haller D (2018) Mitochondrial function — gatekeeper of intestinal epithelial cell homeostasis. Nat Rev Gastroenterol Hepatol 15(8):497–516. 10.1038/s41575-018-0021-x 29844587 10.1038/s41575-018-0021-x

[CR62] Riddell N, Crewther SG (2017) Integrated comparison of GWAS, transcriptome, and proteomics studies highlights similarities in the biological basis of animal and human myopia. Invest Ophthalmol Vis Sci 58(1):660–669. 10.1167/IOVS.16-20618 28135361 10.1167/iovs.16-20618

[CR63] Ringø E, Van Doan H, Lee SH, Soltani M, Hoseinifar SH, Harikrishnan R, Song SK (2020) Probiotics, lactic acid bacteria and bacilli: interesting supplementation for aquaculture. J Appl Microbiol 129(1):116–136. 10.1111/JAM.14628 32141152 10.1111/jam.14628

[CR64] Rocha SDC, Lei P, Morales-Lange B, Mydland LT, Øverland M (2023) From a cell model to a fish trial: Immunomodulatory effects of heat-killed *Lactiplantibacillus plantarum* as a functional ingredient in aquafeeds for salmonids. Front Immunol 14:1125702. 10.3389/fimmu.2023.1125702 36993984 10.3389/fimmu.2023.1125702 PMC10040762

[CR65] Roeselers G, Mittge EK, Stephens WZ, Parichy DM, Cavanaugh CM, Guillemin K, Rawls JF (2011) Evidence for a core gut microbiota in the zebrafish. ISME J 5(10):1595–1608. 10.1038/ISMEJ.2011.38 21472014 10.1038/ismej.2011.38 PMC3176511

[CR66] Rosado D, Xavier R, Severino R, Tavares F, Cable J, Pérez-Losada M (2019) Effects of disease, antibiotic treatment and recovery trajectory on the microbiome of farmed seabass (*Dicentrarchus labrax*). Sci Rep 9(1):1–11. 10.1038/S41598-019-55314-4 31831775 10.1038/s41598-019-55314-4 PMC6908611

[CR67] Sadeghi J, Chaganti SR, Johnson TB, Heath DD (2023) Host species and habitat shape fish-associated bacterial communities: phylosymbiosis between fish and their microbiome. Microbiome 11(1):258. 10.1186/s40168-023-01697-6 37981701 10.1186/s40168-023-01697-6 PMC10658978

[CR68] Sagada G, Wang L, Xu B, Sun Y, Shao Q (2025) Interactive effect of dietary heat-killed *Lactobacillus Plantarum* L-137 and berberine supplementation on intestinal mucosa and microbiota of juvenile black sea bream (*Acanthopagrus Schlegelii*). Probiot Antimicrob Proteins 17(1):419–431. 10.1007/S12602-023-10153-8/10.1007/s12602-023-10153-8 37740880

[CR69] Salminen S, Collado MC, Endo A, Hill C, Lebeer S, Quigley EMM, Sanders ME, Shamir R, Swann JR, Szajewska H, Vinderola G (2021) The international scientific association of probiotics and prebiotics (ISAPP) consensus statement on the definition and scope of postbiotics. Nat Rev Gastroenterol Hepatol 18(9):649–667. 10.1038/s41575-021-00440-6 33948025 10.1038/s41575-021-00440-6 PMC8387231

[CR70] Shi SB, Wu JF, Yang LF, Jiang MG, Gao CM, Jiang CL, Jiang Y (2021) *Crenobacter intestini* sp. nov., isolated from the intestinal tract of Konosirus punctatus. Curr Microbiol 78:1686–1691. 10.1007/s00284-021-02372-5 33683417 10.1007/s00284-021-02372-5

[CR71] Siriyappagouder P, Galindo-Villegas J, Lokesh J, Mulero V, Fernandes JMO, Kiron V (2018) Exposure to yeast shapes the intestinal bacterial community assembly in zebrafish larvae. Front Microbiol 9:397250. 10.3389/FMICB.2018.01868/10.3389/fmicb.2018.01868 PMC6103253 30154775

[CR72] Soliman AM, Barreda DR (2023) The acute inflammatory response of teleost fish. Dev Comp Immunol 146:104731. 10.1016/J.DCI.2023.104731 37196851 10.1016/j.dci.2023.104731

[CR73] Stead JD, Lee H, Williams A, Ramírez SAC, Atlas E, Mennigen JA, O’Brien JM, Yauk C (2025) Gene set enrichment analysis in zebrafish embryos is susceptible to false-positive results in the absence of differentially expressed genes. Bioinform Biol Insights 19:11779322251321071. 10.1177/11779322251321071 40040651 10.1177/11779322251321071 PMC11877468

[CR74] Taddese R, Belzer C, Aalvink S, de Jonge MI, Nagtegaal ID, Dutilh BE, Boleij A (2021) Production of inactivated Gram-positive and Gram-negative species with preserved cellular morphology and integrity. J Microbiol Methods 184:106208. 10.1016/J.MIMET.2021.106208 33766606 10.1016/j.mimet.2021.106208

[CR75] Tapia-Paniagua ST, Chabrillón M, Díaz-Rosales P, de la Banda IG, Lobo C, Balebona MC, Moriñigo MA (2010) Intestinal microbiota diversity of the flat fish *Solea senegalensis* (Kaup, 1858) following probiotic administration. Microb Ecol 60(2):310–319. 10.1007/S00248-010-9680-Z 20556376 10.1007/s00248-010-9680-z

[CR76] Thormar EA, Rasmussen JA, Mathiessen H, Marana MH, Clausen CG, Hansen M, Kodama M, von Gersdorff Jørgensen L, Limborg MT (2024) A zebrafish model to elucidate the impact of host genes on the microbiota. Environ DNA 6(1):e513. 10.1002/edn3.513

[CR77] Ulloa PE, Iturra P, Neira R, Araneda C (2011) Zebrafish as a model organism for nutrition and growth: towards comparative studies of nutritional genomics applied to aquacultured fishes. Rev Fish Biol Fish 21(4):649–666. 10.1007/s11160-011-9203-0

[CR78] Ulloa PE, Medrano JF, Feijoo CG (2014) Zebrafish as animal model for aquaculture nutrition research. Front Genet 5:313. 10.3389/fgene.2014.00313 25309575 10.3389/fgene.2014.00313 PMC4160086

[CR79] Van Nguyen N, Onoda S, Van Khanh T, Hai PD, Trung NT, Hoang L, Koshio S (2019) Evaluation of dietary heat-killed *Lactobacillus plantarum* strain L-137 supplementation on growth performance, immunity and stress resistance of Nile tilapia (*Oreochromis niloticus*). Aquaculture 498:371–379. 10.1016/J.AQUACULTURE.2018.08.081

[CR80] Vásquez-Piñeros MA, Martínez-Lavanchy PM, Jehmlich N, Pieper DH, Rincón CA, Harms H, Junca H, Heipieper HJ (2018) *Delftia* sp. LCW, a strain isolated from a constructed wetland shows novel properties for dimethylphenol isomers degradation. BMC Microbiol 18(1):1–12. 10.1186/S12866-018-1255-Z/30189831 10.1186/s12866-018-1255-z PMC6127914

[CR81] Vaziri ND (2014) Role of dyslipidemia in impairment of energy metabolism, oxidative stress, inflammation and cardiovascular disease in chronic kidney disease. Clin Exp Nephrol 18(2):265–268. 10.1007/S10157-013-0847-Z/23974528 10.1007/s10157-013-0847-z

[CR82] Vinderola G, Sanders ME, Cunningham M, Hill C (2023) Frequently asked questions about the ISAPP postbiotic definition. Front Microbiol 14:1324565. 10.3389/FMICB.2023.1324565/38268705 10.3389/fmicb.2023.1324565 PMC10807003

[CR83] Wang A, Zhang Z, Ding Q, Yang Y, Bindelle J, Ran C, Zhou Z (2021) Intestinal *Cetobacterium* and acetate modify glucose homeostasis via parasympathetic activation in zebrafish. Gut Microbes 13(1):1–15. 10.1080/19490976.2021.1900996 33840371 10.1080/19490976.2021.1900996 PMC8043178

[CR84] Wei Y, Liu J, Wang L, Duan M, Ma Q, Xu H, Liang M (2023) Influence of fish protein hydrolysate on intestinal health and microbial communities in turbot *Scophthalmus maximus*. Aquaculture 576:739827. 10.1016/J.AQUACULTURE.2023.739827

[CR85] Xie S, Wei D, Liu Y, Tian L, Niu J (2022) Dietary fish oil levels modulated lipid metabolism, immune response, intestinal health and salinity stress resistance of juvenile *Penaeus monodon* fed a low fish-meal diet. Anim Feed Sci Technol 289:115321. 10.1016/J.ANIFEEDSCI.2022.115321

[CR86] Yang HL, Xia HQ, Ye YD, Zou WC, Sun YZ (2014) Probiotic *Bacillus pumilus* SE5 shapes the intestinal microbiota and mucosal immunity in grouper *Epinephelus coioides*. Dis Aquat Org 111(2):119–127. 10.3354/DAO02772 10.3354/dao02772 25266899

[CR87] Yu G, Wang LG, Han Y, He Q-Y (2012) Clusterprofiler: an R package for comparing biological themes among gene clusters. OMICS 16(5):284–287 22455463 10.1089/omi.2011.0118 PMC3339379

[CR88] Zhang C, Wang K, Yang L, Liu R, Chu Y, Qin X, Yang P, Yu H (2018) Lipid metabolism in inflammation-related diseases. Analyst 143(19):4526–4536. 10.1039/C8AN01046C 30128447 10.1039/c8an01046c

[CR89] Zhang SQ, Xie CJ, Yao L, Rensing C, Lin H, Liu G-H, Zhou S-G (2024) *Crenobacter oryzisoli* sp. nov., a novel phosphate-solubilizing bacterium isolated from the paddy soil. Arch Microbiol 206:337. 10.1007/s00203-024-04070-9 38954015 10.1007/s00203-024-04070-9

[CR90] Zhong X, Li J, Lu F, Zhang J, Guo L (2022) Application of zebrafish in the study of the gut microbiome. Anim Models Exp Med 5(4):323–336. 10.1002/AME2.12227 10.1002/ame2.12227 PMC9434591 35415967

[CR91] Zou J, Secombes CJ (2016) The function of fish cytokines. Biology 5(2):23. 10.3390/BIOLOGY5020023 27231948 10.3390/biology5020023 PMC4929537

