# Supplementary material for: Vibrio proteolyticus DCF12.2 postbiotic modulates intestinal metabolic and immune pathways in zebrafish
Source: Appl Microbiol Biotechnol. 2026 Apr 22;110:174. doi: 10.1007/s00253-026-13825-x (PMC13236790; doi:10.1007/s00253-026-13825-x)
Supplement: Supplementary file 1 [file 253_2026_13825_MOESM1_ESM.docx]

**Supporting information - Applied Microbiology and Biotechnology**

***Vibrio proteolyticus* DCF12.2 postbiotic modulates gut metabolism without altering microbiota in zebrafish**

**Jorge García-Márquez^1,2,3,^‡, Isabel Cerezo^3,4,^‡, Rafaela Santos^1,2^, Rui Magalhães^1,2^, António Paulo Carvalho^1,2^, Salvador Arijo^3^, Aires Oliva-Teles^1,2^, Cláudia Serra^1,2,^*, Miguel Ángel Moriñigo^1,^***

^1^ Centro Interdisciplinar de Investigação Marinha e Ambiental (CIIMAR), Universidade do Porto, Terminal de Cruzeiros do Porto de Leixões, Av, General Norton de Matos s/n, 4450-208 Matosinhos, Portugal; jgarcia@ciimar.up.pt (J.G.-M.); rafaela.santos@ciimar.up.pt (R.S.); rmagalhaes@ciimar.up.pt (R.M.); apcarval@fc.up.pt (A.P.C.); up201829@up.pt (A.O.-T.); cserra@ciimar.up.pt (C.S.)

^2^ Departamento de Biologia, Faculdade de Ciências, University of Porto, Rua do Campo Alegre s/n, Ed. FC4,

4169-007 Porto, Portugal

^3^ Departamento de Microbiología, Facultad de Ciencias, Universidad de Málaga, Instituto Andaluz de Biotecnología y Desarrollo Azul (IBYDA), Campus Universitario de Teatinos s/n, 29071, Málaga, Spain; cerezoi@uma.es (I.C.); [sarijo@uma.es](mailto:sarijo@uma.es) (S.A.); morinigo@uma.es (M.A.M.)

^4^ Departamento de Biología y Geología, Ceimar-Universidad de Almería, 04120 La Cañada de San Urbano, Almería, Spain

*Correspondence: cserra@ciimar.up.pt (C.S.); [morinigo@uma.es](mailto:morinigo@uma.es) (M.A.M.)

‡These authors have contributed equally to this work.

**Fig. S1.** Principal coordinate analysis (PCoA) of the bacterial communities of the microbiota of zebrafish fed the experimental diets, calculated from the weighted UniFrac distance matrix. Red circles and blue triangles represent the microbiota of each zebrafish individual fed the Control (CTRL) and *V. proteolyticus* DCF12.2 (VP) diets, respectively.

**Fig. S2**. Relative abundance (percentage) at phylum level for individual samples of intestinal microbiota of zebrafish fed Control (CTRL) and ethanol-inactivated *V. proteolyticus* DCF12.2 (VP) diets. < 1% indicates a mean relative abundance of less than 1%. NA represents unassigned taxa.

**Fig. S3.** Relative abundance (percentage) at the genus level for individual samples of intestinal microbiota of zebrafish fed Control (CTRL) and ethanol-inactivated *V. proteolyticus* DCF12.2 (VP) diets. < 1% indicates a mean relative abundance of less than 1%. NA represents unassigned taxa.

**Fig. S4**. Effect size and volcano plots for the differential abundance of functional predictions from PICRUSt2 using ALDEx2 in the intestine of zebrafish fed Control (CTRL) and ethanol-inactivated *V. proteolyticus* DCF12.2 (VP) diets. Blue dots indicate unadjusted *p* values, while red dots represent the Benjamini–Hochberg adjusted *p* values. The grey dashed horizontal line represents the threshold value of *p* = 0.05.

**Fig. S5**. Top 20 of GO Biological process enrichment of upregulated Genes (ORA) in response to the dietary administration of *V. proteolyticus* DCF12.2.

**Fig. S6**. Top 20 of GO Biological process enrichment of downregulated Genes (ORA) in response to the dietary administration of *V. proteolyticus* DCF12.2.

**Fig. S7**. KEGG enrichment of regulated Genes (ORA) in response to the dietary administration of *V. proteolyticus* DCF12.2.

**Table S1.** Assigned ASVs classified at the genus level in the gut microbiota of zebrafish fed the experimental diets. Only total relative abundance > 1% is considered. Different ASVs identified by DESeq2 analysis are highlighted in bold.

| **ASV assigned at genus level** | | **CTRL** | | **VP** | | ***p-*value** |
| --- | --- | --- | --- | --- | --- | --- |
| **ASV number** | **Genus** | **Mean** | **SD** | **Mean** | **SD** |  |
| Seq_59 | *Aeromonas* | 0.21 | 0.42 | 0.75 | 2.25 | n.s. |
| Seq_60 | *Aeromonas* | 0.57 | 0.95 | 0.33 | 0.62 | n.s. |
| Seq_40 | *Aeromonas* | 1.45 | 3.54 | 0.37 | 0.74 | n.s. |
| **Seq_129** | ***Aeromonas*** | **0.00** | **0.00** | **0.14** | **0.22** | **<0.0001** |
| **Seq_48** | ***Aeromonas*** | **0.00** | **0.00** | **1.37** | **2.08** | **<0.0001** |
| Seq_58 | *Aeromonas* | 0.21 | 0.42 | 0.80 | 2.41 | n.s. |
| Seq_257 | *Aeromonas* | 0.03 | 0.08 | 0.01 | 0.03 | n.s. |
| Seq_65 | *Aeromonas* | 0.46 | 0.79 | 0.29 | 0.50 | n.s. |
| Seq_149 | *Aeromonas* | 0.05 | 0.16 | 0.01 | 0.04 | n.s. |
| Seq_136 | *Aeromonas* | 0.07 | 0.20 | 0.03 | 0.05 | n.s. |
| **Seq_131** | ***Aeromonas*** | **0.00** | **0.00** | **0.13** | **0.20** | **<0.0001** |
| **Seq_47** | ***Aeromonas*** | **0.00** | **0.00** | **1.34** | **2.03** | **<0.0001** |
| Seq_44 | *Aeromonas* | 1.35 | 3.28 | 0.34 | 0.67 | n.s. |
| Seq_3 | *Cetobacterium* | 5.98 | 9.16 | 4.92 | 9.77 | n.s. |
| **Seq_10** | ***Cetobacterium*** | **0.00** | **0.00** | **1.82** | **4.63** | **<0.0001** |
| Seq_27 | *Cetobacterium* | 1.09 | 2.30 | 0.56 | 1.67 | n.s. |
| Seq_4 | *Cetobacterium* | 6.49 | 8.75 | 3.19 | 4.97 | n.s. |
| Seq_268 | *Cetobacterium* | 0.00 | 0.00 | 0.03 | 0.07 | n.s. |
| **Seq_12** | ***Cetobacterium*** | **3.02** | **6.31** | **0.63** | **1.90** | **<0.0001** |
| Seq_1 | *Cetobacterium* | 6.44 | 9.90 | 5.31 | 10.56 | n.s. |
| **Seq_37** | ***Cetobacterium*** | **0.00** | **0.00** | **0.39** | **0.82** | **<0.0001** |
| Seq_2 | *Cetobacterium* | 8.06 | 10.82 | 4.02 | 6.28 | n.s. |
| Seq_16 | *Cetobacterium* | 2.43 | 3.07 | 1.80 | 2.93 | n.s. |
| Seq_318 | *Cetobacterium* | 0.00 | 0.00 | 0.03 | 0.06 | n.s. |
| Seq_17 | *Cetobacterium* | 1.90 | 3.33 | 1.89 | 3.76 | n.s. |
| Seq_30 | *Cetobacterium* | 1.08 | 2.30 | 0.54 | 1.62 | n.s. |
| **Seq_11** | ***Cetobacterium*** | **0.00** | **0.00** | **1.87** | **4.64** | **<0.0001** |
| Seq_9 | *Cetobacterium* | 2.96 | 3.72 | 2.26 | 3.75 | n.s. |
| **Seq_15** | ***Cetobacterium*** | **2.85** | **5.96** | **0.62** | **1.86** | **<0.0001** |
| **Seq_36** | ***Cetobacterium*** | **0.00** | **0.00** | **0.38** | **0.81** | **<0.0001** |
| Seq_133 | *Cetobacterium* | 0.00 | 0.00 | 0.08 | 0.17 | n.s. |
| Seq_13 | *Cetobacterium* | 2.01 | 3.54 | 2.06 | 4.11 | n.s. |
| Seq_127 | *Cetobacterium* | 0.00 | 0.00 | 0.07 | 0.15 | n.s. |
| Seq_88 | *Crenobacter* | 0.16 | 0.33 | 0.05 | 0.16 | n.s. |
| Seq_54 | *Crenobacter* | 0.59 | 0.83 | 0.67 | 1.14 | n.s. |
| Seq_66 | *Crenobacter* | 0.29 | 0.52 | 0.20 | 0.42 | n.s. |
| **Seq_85** | ***Crenobacter*** | **0.00** | **0.00** | **0.10** | **0.20** | **<0.0001** |
| Seq_61 | *Crenobacter* | 0.46 | 0.67 | 0.54 | 0.91 | n.s. |
| Seq_80 | *Crenobacter* | 0.18 | 0.38 | 0.06 | 0.19 | n.s. |
| **Seq_91** | ***Crenobacter*** | **0.00** | **0.00** | **0.10** | **0.22** | **<0.0001** |
| Seq_62 | *Crenobacter* | 0.32 | 0.57 | 0.22 | 0.45 | n.s. |
| **Seq_41** | ***Delftia*** | **0.00** | **0.00** | **0.89** | **1.34** | **<0.0001** |
| **Seq_43** | ***Delftia*** | **0.00** | **0.00** | **0.92** | **1.38** | **<0.0001** |
| Seq_55 | *Paucibacter* | 0.44 | 0.79 | 0.33 | 0.66 | n.s. |
| Seq_92 | *Paucibacter* | 0.00 | 0.00 | 0.07 | 0.13 | n.s. |
| Seq_56 | *Paucibacter* | 0.44 | 0.79 | 0.33 | 0.65 | n.s. |
| Seq_46 | *Paucibacter* | 0.92 | 1.42 | 0.92 | 1.54 | n.s. |
| Seq_95 | *Paucibacter* | 0.00 | 0.00 | 0.06 | 0.10 | n.s. |
| Seq_79 | *Paucibacter* | 0.19 | 0.39 | 0.10 | 0.30 | n.s. |
| Seq_78 | *Paucibacter* | 0.18 | 0.37 | 0.10 | 0.31 | n.s. |
| Seq_38 | *Paucibacter* | 1.12 | 1.75 | 1.19 | 1.96 | n.s. |
| Seq_7 | *Plesiomonas* | 5.40 | 10.72 | 2.94 | 8.81 | n.s. |
| Seq_33 | *Plesiomonas* | 1.49 | 2.41 | 0.82 | 1.40 | n.s. |
| Seq_42 | *Plesiomonas* | 1.07 | 1.69 | 0.41 | 0.84 | n.s. |
| Seq_45 | *Plesiomonas* | 0.99 | 1.54 | 0.40 | 0.83 | n.s. |
| **Seq_20** | ***Plesiomonas*** | **0.00** | **0.00** | **1.70** | **3.83** | **<0.0001** |
| **Seq_21** | ***Plesiomonas*** | **0.00** | **0.00** | **1.67** | **3.73** | **<0.0001** |
| Seq_8 | *Plesiomonas* | 5.01 | 9.95 | 2.73 | 8.18 | n.s. |
| Seq_39 | *Plesiomonas* | 1.24 | 2.03 | 0.73 | 1.22 | n.s. |
| **Seq_6** | ***Ralstonia*** | **0.00** | **0.00** | **6.07** | **10.65** | **<0.0001** |
| Seq_366 | *Ralstonia* | 0.03 | 0.07 | 0.02 | 0.06 | n.s. |
| **Seq_5** | ***Ralstonia*** | **0.00** | **0.00** | **6.09** | **10.54** | **<0.0001** |
| Seq_616 | *Ralstonia* | 0.01 | 0.02 | 0.01 | 0.01 | n.s. |
| Seq_31 | *Ralstonia* | 3.59 | 10.76 | 3.66 | 10.94 | n.s. |
| Seq_34 | *Ralstonia* | 2.96 | 8.84 | 3.07 | 9.18 | n.s. |
| Seq_77 | *Shewanella* | 0.19 | 0.29 | 0.12 | 0.23 | n.s. |
| **Seq_87** | ***Shewanella*** | **0.14** | **0.29** | **0.28** | **0.84** | **<0.0001** |
| Seq_247 | *Shewanella* | 0.04 | 0.06 | 0.02 | 0.04 | n.s. |
| **Seq_104** | ***Shewanella*** | **0.00** | **0.00** | **0.18** | **0.44** | **<0.0001** |
| Seq_123 | *Shewanella* | 0.14 | 0.35 | 0.01 | 0.02 | n.s. |
| **Seq_76** | ***Shewanella*** | **0.17** | **0.36** | **0.33** | **0.99** | **<0.0001** |
| Seq_106 | *Shewanella* | 0.00 | 0.00 | 0.16 | 0.44 | n.s. |
| Seq_122 | *Shewanella* | 0.14 | 0.34 | 0.02 | 0.04 | n.s. |
| Seq_83 | *Shewanella* | 0.18 | 0.28 | 0.10 | 0.19 | n.s. |
| Seq_141 | *Shewanella* | 0.00 | 0.00 | 0.02 | 0.05 | n.s. |
| Seq_150 | *Shewanella* | 0.00 | 0.00 | 0.01 | 0.03 | n.s. |
| Seq_90 | *Shewanella* | 0.25 | 0.39 | 0.17 | 0.26 | n.s. |
| Seq_75 | *Shewanella* | 0.31 | 0.46 | 0.21 | 0.33 | n.s. |
| Seq_288 | *Shewanella* | 0.04 | 0.07 | 0.02 | 0.04 | n.s. |
| **Seq_19** | ***Stenotrophomonas*** | **0.00** | **0.00** | **1.80** | **2.96** | **<0.0001** |
| **Seq_18** | ***Stenotrophomonas*** | **0.00** | **0.00** | **1.73** | **2.84** | **<0.0001** |
| **Seq_29** | ***ZOR0006*** | **0.00** | **0.00** | **0.66** | **1.40** | **<0.0001** |
| Seq_52 | *ZOR0006* | 0.46 | 0.91 | 0.16 | 0.49 | n.s. |
| Seq_53 | *ZOR0006* | 0.46 | 0.91 | 0.16 | 0.49 | n.s. |
| Seq_22 | *ZOR0006* | 1.64 | 2.29 | 2.65 | 5.22 | n.s. |
| Seq_24 | *ZOR0006* | 2.39 | 4.92 | 0.74 | 1.82 | n.s. |
| **Seq_32** | ***ZOR0006*** | **0.00** | **0.00** | **0.61** | **1.29** | **<0.0001** |
| Seq_14 | *ZOR0006* | 1.96 | 2.78 | 3.24 | 6.36 | n.s. |
| Seq_23 | *ZOR0006* | 2.48 | 5.19 | 0.75 | 1.82 | n.s. |

n.s., not significant (DESeq2, *p* ≥ 0.05)

**Table S2.** Differentially expressed genes significantly up- and down-regulated in the intestine of zebrafish fed *V. proteolyticus* DCF12.2 (VP) compared with Control (CTRL), including Gene ID, Gene name, Log2 fold change, *p*‑value, adjusted *p*‑value, and regulation tag.

| **Gene ID** | **Gene name** | **Log2 FC** | ***p*-value** | ***p*-adj** | **Regulation tag** |
| --- | --- | --- | --- | --- | --- |
| 797016 | *si:dkeyp-110e4.11* | 6.032 | 0.000 | 0.030 | Up |
| 559931 | *vtg2* | 5.420 | 0.000 | 0.026 | Up |
| 103911912 | *LOC103911912* | 5.311 | 0.000 | 0.001 | Up |
| 101884145 | *LOC101884145* | 5.088 | 0.000 | 0.007 | Up |
| 559475 | *vtg1* | 4.995 | 0.001 | 0.034 | Up |
| 64260 | *vtg5* | 4.831 | 0.001 | 0.044 | Up |
| 415248 | *rcn3* | 4.517 | 0.000 | 0.000 | Up |
| 100536928 | *LOC100536928* | 4.051 | 0.001 | 0.038 | Up |
| 103910098 | *LOC103910098* | 3.799 | 0.000 | 0.000 | Up |
| 101883185 | *LOC101883185* | 3.686 | 0.000 | 0.002 | Up |
| 403045 | *egf* | 3.604 | 0.001 | 0.049 | Up |
| 108190615 | *LOC108190615* | 3.367 | 0.000 | 0.013 | Up |
| 259252 | *esr1* | 2.963 | 0.000 | 0.015 | Up |
| 100005373 | *greb1l* | 2.956 | 0.001 | 0.039 | Up |
| 767641 | *zgc:153219* | 2.504 | 0.001 | 0.047 | Up |
| 792450 | *LOC792450* | 2.498 | 0.001 | 0.048 | Up |
| 110438982 | *LOC110438982* | 2.454 | 0.001 | 0.038 | Up |
| 325939 | *si:dkey-286h2.7* | 1.768 | 0.000 | 0.002 | Up |
| 393308 | *riox2* | 1.757 | 0.000 | 0.010 | Up |
| 100005033 | *si:ch211-243a15.1* | 1.718 | 0.001 | 0.035 | Up |
| 259306 | *cyp2ad2* | 1.632 | 0.000 | 0.018 | Up |
| 100006301 | *si:dkey-204l11.1* | 1.575 | 0.000 | 0.027 | Up |
| 100002095 | *zgc:162707* | 1.448 | 0.000 | 0.031 | Up |
| 556280 | *cyp2u1* | 1.422 | 0.000 | 0.019 | Up |
| 402954 | *cbr4* | 1.363 | 0.001 | 0.037 | Up |
| 393141 | *mycb* | 1.348 | 0.001 | 0.049 | Up |
| 559775 | *lman1* | 1.344 | 0.000 | 0.000 | Up |
| 100536187 | *LOC100536187* | 1.329 | 0.001 | 0.042 | Up |
| 767678 | *cish* | 1.297 | 0.000 | 0.030 | Up |
| 406541 | *slc25a25a* | 1.270 | 0.001 | 0.046 | Up |
| 407663 | *si:ch211-107o10.3* | 1.217 | 0.000 | 0.016 | Up |
| 492560 | *im:7141269* | 1.151 | 0.000 | 0.003 | Up |
| 445176 | *mfsd2ab* | 1.145 | 0.000 | 0.023 | Up |
| 606662 | *plgrkt* | 1.133 | 0.000 | 0.000 | Up |
| 569104 | *acoxl* | 1.117 | 0.000 | 0.027 | Up |
| 565337 | *gltpd2* | 1.087 | 0.000 | 0.029 | Up |
| 558617 | *si:dkey-51e6.1* | 1.052 | 0.000 | 0.001 | Up |
| 436772 | *sec61g* | 1.028 | 0.000 | 0.017 | Up |
| 445059 | *adprh* | 1.016 | 0.001 | 0.035 | Up |
| 406415 | *tmem263* | 1.015 | 0.000 | 0.007 | Up |
| 101883597 | *xcr1b.2* | -1.004 | 0.001 | 0.044 | Down |
| 561683 | *nt5c1bb* | -1.006 | 0.000 | 0.021 | Down |
| 799279 | *LOC799279* | -1.006 | 0.000 | 0.011 | Down |
| 619272 | *zgc:113314* | -1.010 | 0.000 | 0.030 | Down |
| 101885260 | *si:ch73-111e15.1* | -1.013 | 0.000 | 0.006 | Down |
| 561574 | *si:ch211-260b17.6* | -1.018 | 0.000 | 0.028 | Down |
| 110439247 | *LOC110439247* | -1.019 | 0.000 | 0.016 | Down |
| 101885327 | *cd180* | -1.033 | 0.000 | 0.016 | Down |
| 337572 | *ctss2.2* | -1.036 | 0.001 | 0.048 | Down |
| 100148503 | *traf1* | -1.040 | 0.000 | 0.011 | Down |
| 101243556 | *tmem173* | -1.042 | 0.000 | 0.031 | Down |
| 100331104 | *si:ch211-193e13.5* | -1.048 | 0.001 | 0.036 | Down |
| 445212 | *fam49al* | -1.072 | 0.000 | 0.006 | Down |
| 110439013 | *LOC110439013* | -1.074 | 0.000 | 0.006 | Down |
| 100005754 | *ftr35* | -1.082 | 0.001 | 0.038 | Down |
| 431720 | *nr4a1* | -1.098 | 0.000 | 0.002 | Down |
| 100536137 | *si:ch211-241b2.5* | -1.101 | 0.000 | 0.009 | Down |
| 436920 | *egr3* | -1.117 | 0.000 | 0.006 | Down |
| 100000151 | *si:dkey-22o12.2* | -1.120 | 0.001 | 0.040 | Down |
| 798168 | *LOC798168* | -1.120 | 0.000 | 0.003 | Down |
| 101885359 | *si:cabz01030277.1* | -1.121 | 0.001 | 0.032 | Down |
| 368241 | *egr2a* | -1.122 | 0.001 | 0.032 | Down |
| 100331455 | *adap2* | -1.123 | 0.000 | 0.009 | Down |
| 100009643 | *ms4a17a.6* | -1.125 | 0.000 | 0.004 | Down |
| 100334535 | *usp18* | -1.125 | 0.000 | 0.023 | Down |
| 101886216 | *LOC101886216* | -1.130 | 0.000 | 0.009 | Down |
| 100151756 | *fam117ab* | -1.131 | 0.000 | 0.000 | Down |
| 100000903 | *prf1.1* | -1.134 | 0.000 | 0.012 | Down |
| 100334819 | *mcoln3a* | -1.136 | 0.001 | 0.038 | Down |
| 794166 | *ccdc69* | -1.136 | 0.000 | 0.009 | Down |
| 563803 | *ccr12a* | -1.137 | 0.000 | 0.023 | Down |
| 560948 | *zgc:174160* | -1.140 | 0.001 | 0.042 | Down |
| 64603 | *eomesa* | -1.143 | 0.001 | 0.047 | Down |
| 566726 | *pctp* | -1.145 | 0.000 | 0.029 | Down |
| 402884 | *tlr21* | -1.145 | 0.000 | 0.000 | Down |
| 492336 | *anxa3b* | -1.150 | 0.000 | 0.011 | Down |
| 405770 | *il1b* | -1.158 | 0.000 | 0.007 | Down |
| 568267 | *kctd17* | -1.174 | 0.000 | 0.024 | Down |
| 569366 | *tnfaip2b* | -1.186 | 0.000 | 0.025 | Down |
| 792926 | *si:dkey-126g1.7* | -1.186 | 0.000 | 0.024 | Down |
| 556950 | *ppm1j* | -1.189 | 0.000 | 0.031 | Down |
| 795305 | *irg1* | -1.190 | 0.001 | 0.038 | Down |
| 799412 | *trpm2* | -1.190 | 0.000 | 0.006 | Down |
| 103908724 | *LOC103908724* | -1.211 | 0.000 | 0.011 | Down |
| 563836 | *zgc:171566* | -1.215 | 0.000 | 0.022 | Down |
| 795524 | *nitr2a* | -1.218 | 0.000 | 0.028 | Down |
| 100000754 | *ms4a17c.1* | -1.227 | 0.001 | 0.039 | Down |
| 101886073 | *LOC101886073* | -1.239 | 0.000 | 0.004 | Down |
| 100005797 | *slc38a5a* | -1.249 | 0.001 | 0.037 | Down |
| 567444 | *irak3* | -1.259 | 0.000 | 0.000 | Down |
| 100148868 | *si:ch211-71m22.3* | -1.272 | 0.001 | 0.049 | Down |
| 795188 | *gpr25* | -1.275 | 0.001 | 0.036 | Down |
| 555518 | *si:dkey-68o6.6* | -1.275 | 0.000 | 0.003 | Down |
| 321786 | *pttg1ipa* | -1.279 | 0.001 | 0.036 | Down |
| 564030 | *bik* | -1.285 | 0.000 | 0.007 | Down |
| 100535674 | *LOC100535674* | -1.302 | 0.000 | 0.012 | Down |
| 101886353 | *fgfbp1b* | -1.334 | 0.000 | 0.011 | Down |
| 562630 | *LOC562630* | -1.369 | 0.000 | 0.007 | Down |
| 102659287 | *trpm4b.1* | -1.381 | 0.000 | 0.018 | Down |
| 100005849 | *rhov* | -1.402 | 0.000 | 0.017 | Down |
| 570584 | *moxd1* | -1.406 | 0.000 | 0.009 | Down |
| 792519 | *adrb3b* | -1.418 | 0.001 | 0.035 | Down |
| 110437762 |  | -1.431 | 0.001 | 0.048 | Down |
| 100537501 | *si:ch73-380l3.3* | -1.438 | 0.001 | 0.042 | Down |
| 555812 | *ucp2* | -1.440 | 0.000 | 0.023 | Down |
| 100134935 | *csf3r* | -1.454 | 0.000 | 0.020 | Down |
| 556374 | *si:dkey-102c8.3* | -1.455 | 0.000 | 0.027 | Down |
| 571165 | *foxp3a* | -1.468 | 0.000 | 0.003 | Down |
| 566318 | *si:ch211-239f4.1* | -1.477 | 0.000 | 0.025 | Down |
| 101886000 | *LOC101886000* | -1.504 | 0.000 | 0.017 | Down |
| 564134 | *arpp21* | -1.526 | 0.001 | 0.043 | Down |
| 101883802 | *LOC101883802* | -1.528 | 0.000 | 0.017 | Down |
| 100001138 | *si:ch211-165b19.8* | -1.531 | 0.001 | 0.043 | Down |
| 100000938 | *cass4* | -1.533 | 0.000 | 0.002 | Down |
| 404039 | *npsn* | -1.549 | 0.000 | 0.022 | Down |
| 103910580 |  | -1.567 | 0.001 | 0.047 | Down |
| 108180146 | *LOC108180146* | -1.568 | 0.001 | 0.036 | Down |
| 565384 | *snap91* | -1.580 | 0.000 | 0.019 | Down |
| 571647 | *mhc1zja* | -1.580 | 0.000 | 0.008 | Down |
| 100034480 | *si:ch211-188c18.1* | -1.596 | 0.001 | 0.044 | Down |
| 103910871 | *LOC103910871* | -1.609 | 0.000 | 0.008 | Down |
| 797322 | *camta1a* | -1.628 | 0.000 | 0.011 | Down |
| 565910 | *creb5b* | -1.637 | 0.000 | 0.031 | Down |
| 568750 | *trim108* | -1.650 | 0.000 | 0.011 | Down |
| 100331729 | *tmem176l.4* | -1.650 | 0.000 | 0.011 | Down |
| 565873 | *cep152* | -1.650 | 0.001 | 0.044 | Down |
| 794185 | *sftpba* | -1.700 | 0.000 | 0.018 | Down |
| 557185 | *slc25a35* | -1.722 | 0.000 | 0.013 | Down |
| 570021 | *LOC570021* | -1.734 | 0.000 | 0.007 | Down |
| 553238 | *esyt3* | -1.740 | 0.001 | 0.033 | Down |
| 567257 | *f3a* | -1.740 | 0.000 | 0.020 | Down |
| 100150280 | *LOC100150280* | -1.745 | 0.000 | 0.010 | Down |
| 393311 | *plekhf1* | -1.764 | 0.000 | 0.015 | Down |
| 100884133 | *dicp1.5-6* | -1.770 | 0.000 | 0.006 | Down |
| 100333959 | *si:ch211-233g6.2* | -1.781 | 0.000 | 0.020 | Down |
| 566550 | *si:ch211-260p9.3* | -1.791 | 0.000 | 0.003 | Down |
| 100534935 | *si:ch211-197h24.8* | -1.793 | 0.000 | 0.002 | Down |
| 100005096 | *LOC100005096* | -1.826 | 0.000 | 0.025 | Down |
| 394003 | *cpne5b* | -1.833 | 0.001 | 0.037 | Down |
| 100000044 | *si:dkey-100n10.2* | -1.887 | 0.000 | 0.012 | Down |
| 100006896 | *mmp13b* | -1.890 | 0.000 | 0.004 | Down |
| 100000986 | *gzm3* | -1.920 | 0.000 | 0.013 | Down |
| 100330638 | *mslnb* | -1.941 | 0.001 | 0.033 | Down |
| 570251 | *LOC570251* | -1.959 | 0.000 | 0.001 | Down |
| 337514 | *mpx* | -1.973 | 0.001 | 0.040 | Down |
| 81581 | *cldnb* | -2.028 | 0.000 | 0.030 | Down |
| 100329848 | *LOC100329848* | -2.029 | 0.000 | 0.001 | Down |
| 393380 | *tspan35* | -2.056 | 0.000 | 0.030 | Down |
| 436948 | *ccr10* | -2.083 | 0.000 | 0.005 | Down |
| 110437969 | *LOC110437969* | -2.091 | 0.000 | 0.024 | Down |
| 559657 | *fam129aa* | -2.120 | 0.001 | 0.046 | Down |
| 100000812 | *si:dkey-172o19.2* | -2.165 | 0.000 | 0.013 | Down |
| 100537421 | *cxcr5* | -2.171 | 0.000 | 0.002 | Down |
| 101883831 | *rnf223* | -2.181 | 0.000 | 0.007 | Down |
| 641321 | *snap23.2* | -2.186 | 0.000 | 0.006 | Down |
| 106144554 | *nkl.2* | -2.225 | 0.001 | 0.034 | Down |
| 108179235 | *si:dkey-42l23.5* | -2.283 | 0.000 | 0.028 | Down |
| 101885005 | *si:ch73-380l3.4* | -2.284 | 0.000 | 0.000 | Down |
| 561111 | *si:dkey-78l4.2* | -2.422 | 0.001 | 0.037 | Down |
| 100150269 | *hecw2a* | -2.478 | 0.001 | 0.039 | Down |
| 793448 | *tgm1l3* | -2.482 | 0.001 | 0.036 | Down |
| 100002785 | *ms4a17a.8* | -2.566 | 0.000 | 0.022 | Down |
| 100536667 | *LOC100536667* | -2.626 | 0.001 | 0.037 | Down |
| 100331195 | *il17rel* | -2.741 | 0.001 | 0.039 | Down |
| 101882982 | *LOC101882982* | -2.759 | 0.000 | 0.023 | Down |
| 100148735 | *LOC100148735* | -2.791 | 0.001 | 0.039 | Down |
| 100007383 | *dicp1.1* | -2.800 | 0.001 | 0.039 | Down |
| 100141339 | *zgc:171509* | -2.823 | 0.000 | 0.009 | Down |
| 555569 | *otogl* | -2.833 | 0.000 | 0.022 | Down |
| 100034647 | *krt17* | -2.835 | 0.001 | 0.038 | Down |
| 100004287 | *slc5a3a* | -2.846 | 0.000 | 0.001 | Down |
| 100330283 | *LOC100330283* | -2.875 | 0.000 | 0.030 | Down |
| 324942 | *grhl1* | -2.922 | 0.000 | 0.008 | Down |
| 100005948 | *LOC100005948* | -3.055 | 0.000 | 0.025 | Down |
| 100000389 | *si:ch73-347e22.8* | -3.134 | 0.000 | 0.025 | Down |
| 110439020 | *LOC110439020* | -3.142 | 0.000 | 0.015 | Down |
| 108182769 | *LOC108182769* | -3.217 | 0.001 | 0.033 | Down |
| 561765 | *noxo1b* | -3.319 | 0.000 | 0.011 | Down |
| 564665 | *crlf1b* | -3.321 | 0.000 | 0.010 | Down |
| 553296 | *hrc* | -3.436 | 0.001 | 0.047 | Down |
| 101884914 | *si:ch211-170a17.1* | -3.440 | 0.001 | 0.049 | Down |
| 110439067 | *LOC110439067* | -3.503 | 0.001 | 0.032 | Down |
| 100333859 | *si:dkey-162h11.3* | -3.841 | 0.000 | 0.013 | Down |
| 101882922 | *timp4.1* | -3.906 | 0.000 | 0.007 | Down |
| 100004199 | *LOC100004199* | -3.971 | 0.000 | 0.000 | Down |
| 110437745 | *LOC110437745* | -3.999 | 0.000 | 0.018 | Down |
| 335563 | *nkl.4* | -4.011 | 0.000 | 0.001 | Down |
| 447832 | *capn3a* | -4.113 | 0.001 | 0.033 | Down |
| 568018 | *rpgrip1* | -4.307 | 0.000 | 0.018 | Down |
| 553515 | *si:ch211-95j8.2* | -4.351 | 0.000 | 0.031 | Down |
| 100034650 | *si:dkey-21e2.7* | -4.636 | 0.000 | 0.003 | Down |
| 569718 | *si:dkey-65b12.6* | -4.784 | 0.000 | 0.006 | Down |
| 110438862 | *LOC110438862* | -4.844 | 0.000 | 0.004 | Down |
| 100884151 | *dicp1.7* | -4.851 | 0.000 | 0.002 | Down |
| 100147849 | *LOC100147849* | -4.876 | 0.000 | 0.010 | Down |
| 572528 | *arid6* | -4.915 | 0.000 | 0.030 | Down |
| 108190590 | *LOC108190590* | -5.026 | 0.001 | 0.043 | Down |
| 81584 | *cldne* | -5.033 | 0.000 | 0.008 | Down |
| 393394 | *btr30* | -5.723 | 0.000 | 0.022 | Down |
| 100006550 | *zgc:163079* | -6.415 | 0.001 | 0.031 | Down |
| 553353 | *si:ch211-202f3.3* | -6.751 | 0.000 | 0.004 | Down |
